# International incidence and mortality trends of liver cancer: a global profile

**DOI:** 10.1038/srep45846

**Published:** 2017-03-31

**Authors:** Martin C. S. Wong, Johnny Y. Jiang, William B Goggins, Miaoyin Liang, Yuan Fang, Franklin D. H. Fung, Colette Leung, Harry H. X. Wang, Grace L. H. Wong, Vincent W.S. Wong, Henry L. Y. Chan

**Affiliations:** 1 School of Public Health and Primary Care, Faculty of Medicine, Chinese University of Hong Kong, Prince of Wales Hospital, Shatin, New Territories, Hong Kong; 2 Institute of Digestive Disease, Faculty of Medicine, Chinese University of Hong Kong, Hong Kong; 3 Peking Union School of Public Health, Chinese Academy of Medical Sciences and Peking Union Medical College, China; 4 School of Public Health, Sun Yat-Sen University, Guangzhou, 510080, P.R. China; 5 General Practice and Primary Care, Institute of Health and Wellbeing, University of Glasgow, Glasgow G12 9LX, UK; 6 Department of Medicine and Therapeutics, Faculty of Medicine, Chinese University of Hong Kong, Hong Kong; 7 State Key Laboratory of Digestive Disease, Faculty of Medicine, Chinese University of Hong Kong, Hong Kong.

## Abstract

We examined the global incidence and mortality rates of liver cancer, and evaluated the association between incidence/mortality and socioeconomic development (Human Development Index [HDI] and Gross Domestic Product [GDP]) using linear regression analysis. The average annual percent change (AAPC) of the trends was evaluated from join-point regression analysis. The global incidence of liver cancer varied widely by nine-fold, and was negatively correlated with HDI (men: r = −0.232, p = 0.003; women: r = −0.369, p < 0.001) and GDP per capita (men: r = −0.164, p = 0.036; women: r = −0.212, p = 0.007). Its mortality showed a similarly negative correlation with both indices. The greatest incidence rise in men was observed in Poland (AAPC = 17.5, 95% C.I. = 5.6, 30.9) and Brazil (AAPC = 13.2, 95% C.I. = 5.9, 21.0), whereas Germany (AAPC = 6.6, 95% C.I = 2.0, 11.5) and Norway (AAPC = 6.5, 95% C.I. = 3.2, 10.0) had the greatest increase in women. The mortality rates paralleled the incidence rates in most countries. For mortality, Malta (AAPC = 11.5, 95% C.I. = 3.9, 19.8), Australia (AAPC = 6.8, 95% C.I. = 2.2, 11.5) and Norway (APCC = 5.6, 95% C.I. = 2.8, 8.5) reported the biggest increase among men; whilst Australia (AAPC = 13.4, 95% C.I. = 7.8, 19.4) and Singapore (AAPC = 7.7, 95% C.I. = 4.1, 11.5) showed the most prominent rise among women. These epidemiological data identified countries with potentially increasing trends of liver cancer for preventive actions.

Globally, liver cancer is the fifth commonest cancer in 2012, accounting for 9.1% of all cancer deaths worldwide^1^. The global disease burden attributable to this cancer induced a substantial number of years of life lost^2^. Owing to its extremely aggressive nature and poor survival rate^3^, it remains an important public health issue worldwide.

Most liver cancer (83%) was diagnosed in less well developed nations^1^. The vast majority (75–90%) of primary liver cancers are hepatocellular carcinomas (HCCs), with intrahepatic cholangiocarcinoma (ICC) accounting for most of the other cancer subtypes^4^. The recognized risk factors for HCC include chronic hepatitis B virus (HBV) and hepatitis C virus (HCV) infection, exposure to dietary aflatoxin, fatty liver disease, alcohol-induced cirrhosis, obesity, smoking, diabetes, and iron overload^4^,^5^,^6^,^7^. Two important risk factors for ICC include chronic liver fluke infestation and cirrhosis^8^,^9^ he former making ICC the most common subtype of liver cancer diagnosed in Thailand due to its high prevalence of liver fluke^4^.

Since a significant number of risk factors of liver cancer are modifiable, there is a strong prospect to reduce its incidence and mortality by preventive strategies such as lifestyle modification and hepatitis immunization^10^. Some of its etiological factors, including hepatitis infection and cirrhosis, are easily detectable by screening which can minimize development of liver cancer. Hence, it is crucial to understand its epidemiology with respect to its global pattern and trends. Previous studies describing the international trends of liver cancer were based on figures from registries in late 1990s to early 2000s; did not take into account the socioeconomic development of each country when comparisons were made; and depended on comparison among countries in a single calendar year^4^,^10^,^11^. There have also been areas of controversy, including the differential effect of socioeconomic status on the risk of developing liver cancer as an important knowledge gap. Evaluating and analyzing the patterns and temporal trends of this cancer could quantify geographical variation, identify high-risk populations, delineate the extent of preventive strategies implemented, and might provide further insights into disease etiology. These epidemiological data could also be linked to the future prospects of cancer prevention and possibly screening strategies for policy-makers.

This study aims to delineate the patterns and temporal trends of liver cancer in as many countries as available, based on data from high quality cancer registries. We also tested the *a priori* hypothesis that the incidence and mortality of liver cancer, respectively, were associated with differences in socioeconomic development and productivity across different countries.

## Methods

### Source of Data

The incidence and mortality estimates for liver cancer (ICD-10 C22) were retrieved from the GLOBOCAN database for 184 countries in 2012^1^. We made reference to a recent analysis of epidemiological data on colorectal cancer and used similar methodology to evaluate the patterns and trends of liver cancer^12^,^13^. We obtained data on the Human Development Index (HDI) and Gross Domestic Product (GPD) for each country in 2012 from the United Nations Human Development Report^14^. HDI is a composite index of life expectancy, education period, and income per capita indicators, and is perceived as “an index of potential human development”^14^. To examine time trends, information was retrieved from different sources where at least 15 consecutive years of data could be obtained. We included data for countries where both incidence and mortality figures were available. For incidence figures, we extracted high-quality national population-based cancer registries from the *Cancer Incidence in Five Continents* (CI5) series Volumes I-X^15^. To include incidence data for more recent years, we also utilized publicly available information from the U.S.^16^, European countries^17^,^18^,^19^, Australia^20^ and the New Zealand^21^. The incidence data for liver cancer were allocated into different categories according to the International Classification of Diseases 10^th^ revision (ICD-10 C22), whereas mortality data were categorized based on the ICD 9^th^ (155) up to 1991 and 10^th^ version (C22) thereafter^22^. When there are duplicates of incidence or mortality figures obtained from the CI5 and the regional registries, data from the national registries were used in the analysis as they provided more updated figures.

For mortality data, we made reference to the WHO mortality data series where data quality attained criteria of medium level or above^23^, which resulted in data with extensive coverage as well as high accuracy and completeness. Death certificates acted as the primary data source, covering around 30% of the world population, and were compiled by the International Agency for Research on Cancer (IARC) as part of the WHO mortality database. We adopted age-standardized rate (ASR) using the world standard population^24^. Similar to the IARC, we defined more developed countries as all regions of Europe plus Northern America, Australia/New Zealand and Japan, and less developed regions as all regions of Africa, Asia (excluding Japan), Latin America and the Caribbean, Melanesia, Micronesia and Polynesia^1^. From these databases, there were a total of 38 countries where data were available for analysis of incidence and mortality trends.

### Statistical Analysis

We employed joinpoint regression analysis to examine the incidence and mortality trends^25^, using the joinpoint statistical software version 3.4. This technique fits a series of joined straight lines to the trend of ASR^25^. Logarithmic transformation of the rates was performed with computation of the standard errors based on binomial approximation. We specified a maximum number of three joinpoints as analysis options, as in a previous similar study^12^. To determine the direction and magnitude of the recent trends, the average annual percent change (AAPC) and the respective 95% confidence intervals were evaluated for the last available 10 years. The AAPC was calculated as a geometrically weighted average of the various APCs from the joinpoint regression analysis, with weights being equivalent to the length of each segment during the specified time interval^26^. The statistical significance of AAPC was ascertained comparing its magnitude with zero, and all insignificant AAPCs were regarded as having “stable trend”.

The ASRs were plotted against the HDI and GDP per capita, respectively. The HDI was divided into four distinct categories, including low ( ≤ 0.534), medium (0.534 < HDI ≤ 0.710), high (0.710 < HDI ≤ 0.796) and very high (HDL > 0.796) based on the Human Development Report published in 2012^14^. Simple linear regression and correlation coefficients were employed to examine their associations and the goodness-of-fit. All p values < 0.05 were regarded as statistically significant.

## Results

### Incidence and mortality of liver cancer in 2012

A total of 782,451 new cases of liver cancer and 745,533 related deaths were estimated in 2012 (Tables 1 and 2). Approximately 95% of the total incidence and 96% of all mortality occurred in less developed regions. The ratio between the ASR of incidence and mortality was higher in more developed countries than less developed ones in both male (1.21 vs. 1.05) and female (1.08 vs. 1.03). Among all continents, North America and Southern Europe had the highest incidence to mortality ratios. The incidence rates of liver cancer varied more than nine-fold worldwide in 2012. Among men, the highest were found in Eastern Asia (ASR 31.9 per 100,000), South-Eastern Asia (22.2), Northern Africa (18.0) and Western Africa (16.4), and the lowest were reported in South-Central Asia (3.7), Eastern Africa (4.9) and Western Asia (5.0) (Table 1). Among women, the highest were found in Eastern Asia (ASR 10.2 per 100,000), Western Africa (8.1), Melanesia (7.6) and South-Eastern Asia (7.2), and the lowest were reported in Micronesia/Polynesia (1.4), Northern Europe (1.8), Central and Eastern Europe (2.0) and South-Eastern Asia (2.1) (Table 2).

The mortality rates of liver cancer varied by more than eight-fold worldwide in 2012. In men, the highest death rates were reported in the Eastern Asia (29.9), South-Eastern Asia (21.4) and Northern Africa (17.4); whilst in women, the highest mortality was also reported in these three regions (ASR mortality = 9.6, 6.8 and 7.7 per 100,000, respectively). The lowest mortality rates were found in South-Central Asia (3.6), Northern Europe (4.0), and Eastern Africa (4.6) in men. For women, Northern Europe (1.8), Australia/New Zealand (2.0), Western Europe (2.1) and South-Central Asia (2.1) reported the lowest mortality rates. Countries having the highest incidence to mortality ratios in men included North America (1.39), Southern Europe (1.27) and Western Europe (1.23), and the ratios were the highest for women in North America (1.17), Southern Europe (1.16) and Central America (1.08).

### The relationship between incidence/mortality of liver cancer and socioeconomic development

**Figure 1:**
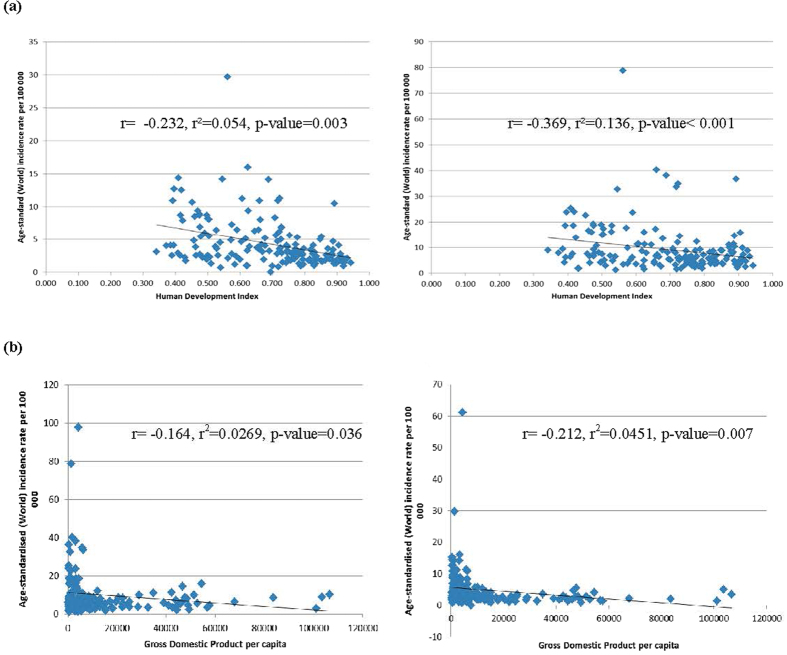
(**a**) Correlation between age-standardised incidence of liver cancer and Human Development Index (HDI) in men (left) and women (right); (**b**). Correlation between age-standardised incidence of liver cancer and Gross Domestic Product (GDP) per capita in men (left) and women (right).

**Figure 2:**
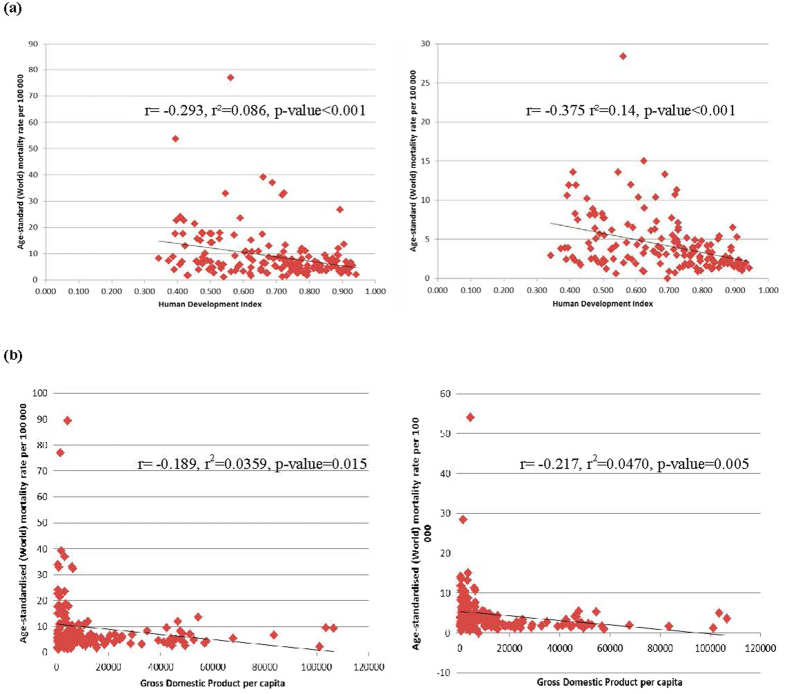
(**a**) Correlation between age-standardised mortality of liver cancer and Human Development Index (HDI) in men (left) and women (right); (**b**). Correlation between age-standardised mortality of liver cancer and Gross Domestic Product (GDP) per capita in men (left) and women (right).

The incidence of liver cancer decreased with higher levels of HDI in men (r^2^ = 0.054, r = −0.232, p = 0.003) and women (r^2^ = 0.136, r = −0.369, p < 0.001), and the same finding was observed for its correlation with GDP per capita (r^2^ = 0.0269, r = −0.164, p = 0.036 and r^2^ = 0.0451, r = −0.212, p = 0.007 for men and women, respectively). For mortality, negative correlations with socioeconomic development were also observed for both HDI (r^2^ = 0.086, r = −0.293, p < 0.001 for men and r^2^ = 0.14, r = −0.375, p < 0.001 for women) and GDP per capita (r^2^ = 0.0359, r = −0.189, p = 0.015 for men and r^2^ = 0.0470, r = −0.217, p = 0.005 for women) (Figs 1a,b and 2a,b).

### Trends in incidence and mortality from liver cancer

Supplementary Figure 1 depicts the temporal trends of incidence and mortality of liver cancer in 38 countries/regions according to gender and countries. Among men, there were a total of 14 countries with increasing incidence trends, 2 countries with decreasing incidence, and 22 countries with stable incidence; whilst for women, there were 8 countries with increasing incidence, 27 countries with stable incidence and 3 with decreasing incidence. For mortality, there were 12 countries with increasing trend, 8 countries with decreasing trend and 16 countries with stable mortality trends in men. There were 8 countries with increasing mortality, 8 countries with decreasing mortality and 20 countries with stable mortality in women. Supplementary Figures 2 and 3 shows the findings from the joinpoint regression analysis. We highlighted the countries with more prominent AAPCs according to the respective continents:

#### Latin America and the Caribbean

**Figure 3:**
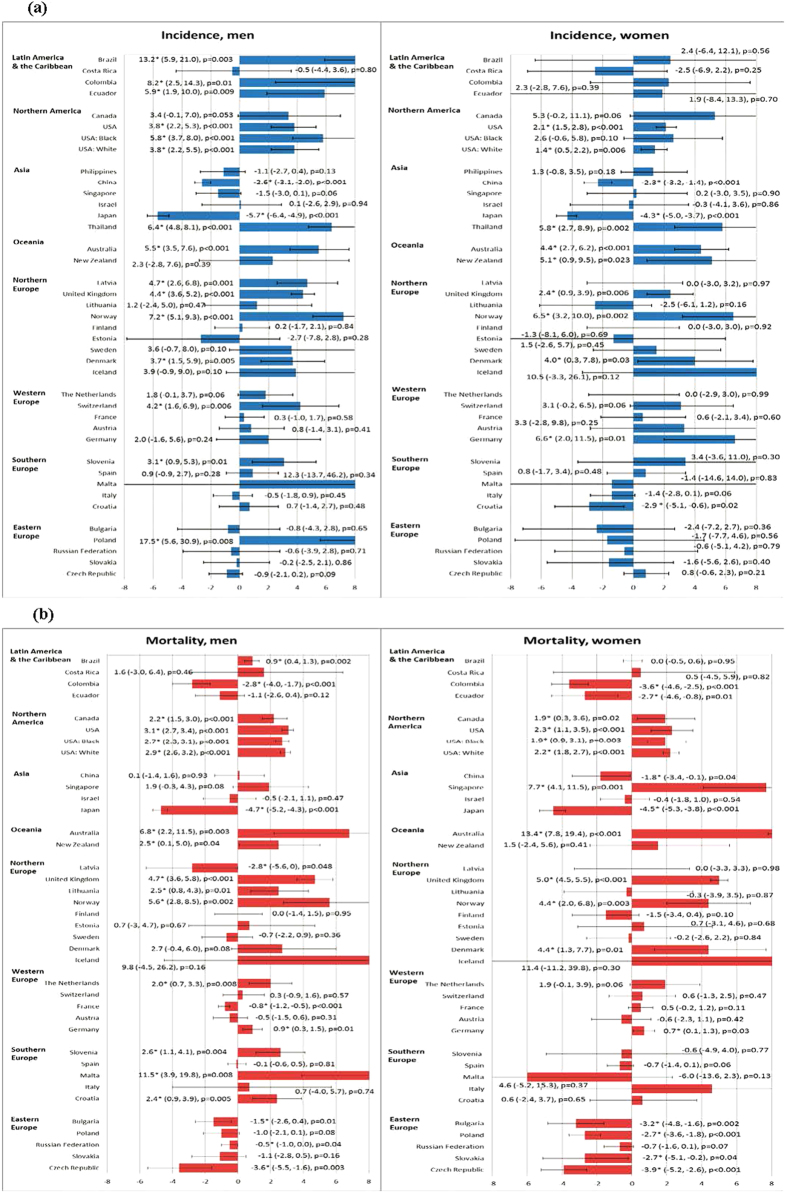
(**a**) The Average Annual Percent Change (AAPC) of liver cancer incidence in men (left) and women (right) in the most recent 10 years; (**b**). The Average Annual Percent Change (AAPC) of liver cancer mortality in men (left) and women (right) in the most recent 10 years.

Brazil (AAPC = 13.2, 95% C.I. = 5.9, 21.0), Colombia (AAPC = 8.2, 95% C.I. = 2.5, 14.3) and Ecuador (AAPC = 5.9, 95% C.I. = 1.9, 10.0) showed an increase in incidence among men, whilst all countries had stable incidence trends in women (Fig. 3a). The ASRs of mortality reported an increase in Brazil (AAPC 0.9, 95% C.I. 0.4, 1.3) and decrease in Colombia in men (AAPC = −2.8, 95% C.I. = −4,0–1.7). Colombia (AAPC = −3.6, 95% C.I. = −4.6, −2.5) and Ecuador (AAPC = −2.7, 95% C.I. = −4.6, −0.8) had significant reduction in mortality among women (Fig. 3b).

#### Northern America

An increase in incidence was observed in male (AAPC = 3.8, 95% C.I. = 2.2, 5.3) and female (AAPC = 2.1, 95% C.I. = 1.5, 2.8) Americans. A slight increase in mortality was reported in the US (AAPC = 3.1, 95% C.I. = 2.7, 3.4 in men; AAPC = 2.3, 95% C.I. = 1.1, 3.5 in women) and Canada (AAPC = 2.2, 95% C.I. = 1.5, 3.0 in men; AAPC = 1.9, 95% C.I. = 0.3, 3.6 in women).

#### Asia

China (AAPC = −2.6, 95% C.I. = −3.1, −2.0) and Japan (AAPC = −5.7, 95% C.I. = −6.4, −4.9) showed a reduction in incidence in men, and these two countries also reported a decline in women (China: AAPC = −2.3, 95% C.I. = −3.2, −1.4; Japan: AAPC = −4.3, 95% C.I. = −5.0, −3.7). Thailand reported an increase in incidence in both men (AAPC = 6.4, 95% C.I. = 4.8, 8.1) and women (AAPC = 5.8, 95% C.I. = 2.7, 8.9). There was a substantial rise in mortality in the Singapore in women (AAPC = 7.7, 95% C.I. = 4.1, 11.5), whereas mortality rates in Japan dropped in both genders.

#### Oceania

There was a slight increase in incidence in Australia (AAPC = 5.5, 95% C.I. = 3.5, 7.6) among men, and Australia among women (AAPC = 4.4, 95% C.I. = 2.7, 6.2). Similarly for mortality, increases were also reported in Australia among men (AAPC = 6.8, 95% C.I. = 2.2, 11.5) and women (AAPC = 13.4, 95% C.I. = 7.8,19.4).

#### Northern Europe

Norway (AAPC = 7.2, 95% C.I. = 5.1, 9.3), Latvia (AAPC = 4.7, 95% C.I. = 2.6, 6.8), United Kingdom (AAPC = 4.4, 95% C.I. = 3.6, 5.2) and Denmark (AAPC = 3.7, 95% C.I. = 1.5, 5.9) were countries that reported an increase in incidence among men. Norway (AAPC = 6.5, 95% C.I. = 3.2, 10.0), Denmark (AAPC = 4,0 95% C.I. = 0.3, 7.8) and United Kingdom (AAPC = 2.4, 95% C.I. = 0.9, 3.9) had incidence increase among women. Norway (AAPC = 5.6, 95% C.I. = 2.8, 8.5), the United Kingdom (AAPC = 4.7, 95% C.I. = 3.6, 5.8) and Lithuania (AAPC = 2.5, 95% C.I. = 0.8, 4.3) reported increase in mortality among men, and the former two also showed an increase in mortality among women.

#### Western Europe

Switzerland showed an increase in incidence among men (AAPC = 4.2, 95% C.I. = 1.6, 6.9) and Germany reported increase in incidence in women (AAPC = 6.6, 95% C.I. = 2.0, 11.5). The Netherlands showed an increase in mortality in men (AAPC = 2.0, 95% C.I. = 0.7, 3.3), and Germany reported an increase in mortality among men (AAPC = 0.9, 95% C.I. = 0.3, 1.5) and women (AAPC = 0.7, 95% C.I. = 0.1, 1.3).

#### Southern Europe

Slovenia (AAPC = 3.1, 95% C.I. = 0.9, 5.3) was the only country that reported rise in incidence among men, whilst Croatia showed a decline in incidence among women (AAPC = −2.9, 95% C.I. = −5.1, −0.6). Malta (AAPC = 11.5, 95% C.I. = 3.9, 19.8), Slovenia (AAPC = 2.6, 95% C.I. = 1.1, 4.1) and Croatia (AAPC = 2.4, 95% C.I. = 0.9, 3.9) reported increase in mortality in men.

#### Eastern Europe

The incidence trends in men and women were all stable, whilst the majority of countries in this continent reported a decline in mortality in both men and women.

## Discussion

### Summary of the Major Findings

This study presented a comprehensive epidemiological analysis of the global profiles of liver cancer incidence and mortality based on high quality data. Geographical variations in its incidence and mortality were substantial - both between and within continents. As of 2012, Eastern Asia, South-Eastern Asia and Northern Africa suffered from the highest incidence in both genders and highest mortality in women. Eastern Asia, Western Africa and Melanesia reported the highest mortality in men. The highest incidence to mortality ratio was found in Northern America and Southern Europe for both men and women. It was found that countries with higher levels of HDI and GDP per capita reported lower incidence and mortality rates of liver cancer. Taking into account the average change of incidence in the previous 10 years, the most remarkable observation included the increase in incidence in Brazil, Colombia and Thailand in men, as well as Brazil and Germany in women. There were rises in mortality rates in Malta, Norway and the United Kingdom in men, and very substantial increase in mortality in Singapore and Denmark in women. Irrespective of gender, a substantial reduction in mortality rate was observed for Japan and Czech Republic. Most countries included in the analysis presented insignificant changes in incidence and mortality trends, with relatively wide 95% confidence intervals.

The results from this study in general corroborated the findings of previous observations^27^,^28^ - where the incidence and mortality were increasing in countries with low incidence of liver cancer, such as Latin America (Brazil), Northern Europe (Norway, UK, Denmark) and Western Europe (Germany). As highlighted by Torre and colleagues, this phenomenon has been attributed to an increase in prevalence of HCV infection in some countries due to injection drug abuse in the 1960s to 1970s^28^. Some studies suggested that escalating rates of obesity and type II diabetes in these relatively well developed countries may be contributory^3^,^29^. On the other hand, the incidence and mortality rates of liver cancer were found to be declining in countries that had higher incidence, such as Japan and China. This observation has been explained by the reduction in aflatoxin exposure and infection with HBV in China due to immunization and other population-based cancer prevention programmes^30^,^31^. The lower incidence rates in Japan could be attributed to reduction in chronic schistosomiasis infection, as well as HCV infection via more hygienic blood donation practices and implementation of policies that deterred intravenous drug use^30^,^32^. Other factors that might change the incidence and mortality of liver cancer include the formulation of country-specific preventive strategies, like reinforcement of lifestyle changes (e.g. smoking and alcohol drinking); community-based health promotion initiatives (e.g. prevention of liver fluke infestations from water sources via education; needle exchange programmes for intravenous drug users); environmental modifications (e.g. improving storage of grains and crop substitution to prevent aflatoxin contamination); and improved medical interventions (e.g. new antiviral therapies for those with acute HCV and chronic HBV or HCV infections; drug treatment for liver fluke)^27^,^33^,^34^,^35^.

We found that countries with higher HDI and GDP per capita had lower incidence and mortality of liver cancer from correlation analysis. Apart from racial and ethnic differences, lower socioeconomic status and poverty have been associated with risk factors for HCC, including diabetes^36^, metabolic syndrome^37^, obesity^38^, alcoholism^39^, HBV^40^, and HCV infection^41^,^42^,^43^. In addition, lower educational attainment has been linked to higher risk of viral hepatitis infection, alcoholism and hepatic inflammation^44^. Changes in some of these risk factors such as alcohol drinking per capita, HDI and GDP per capita might potentially influence the AAPC in each country, and future studies should explore their interrelationships.

This study is the first evaluation of the incidence and mortality trends of liver cancer on a global scale. It presented and analyzed the most up-to-date epidemiological data on this important cancer, and quantified the geographical variations as well as trends in its incidence and mortality using data of high validity, completeness and comparability. We also adopted figures on national mortality that fulfilled criteria attaining at least WHO-defined medium levels of coverage and completeness. The IARCs estimation methods have been further refined in more recent years to take into account the increasing availability and quality of the source data^45^. Nevertheless, some limitations should be addressed. Firstly, failure or under-reporting of cancer diagnosis could lead to bias in cancer registration especially in relatively less-developed nations. Figures in regional cancer registries could be underestimated owing to limited local facilities. On the contrary, in countries where estimates were based on a single cancer registry in more urbanized, resource privileged areas, the presented figures could be an overestimation if the countries consist of extensive rural populations. In addition, only one-third and one-fifth of the world’s countries, respectively, reported incidence and mortality data of high quality. As a result, the incidence and mortality data are constrained with respect to geographical coverage, in particular the resource-deprived countries. It should be noted that the correlation coefficients are not high (ranging from −0.189 to −0.315), and cautions are needed in interpreting the correlation between HDI/GDP and incidence/mortality rates. Lastly, ecological fallacy is an inherent limitation of the present study, where correlations performed in this analysis were derived from group variables.

The incidence and mortality rates of liver cancer increased in many countries that had low rates, although a few countries used to have high rates reported declining incidence trends. Future studies should explore the underlying reasons for these epidemiological trends, which could offer further insights into the specific etiological factors of liver cancer. In addition, the impact of socioeconomic development on the incidence and mortality of liver cancer is of interest, and should be evaluated in future longitudinal studies.

## Additional Information

**How to cite this article:** Wong, M. C. S. *et al*. International incidence and mortality trends of liver cancer: a global profile. *Sci. Rep.* **7**, 45846; doi: 10.1038/srep45846 (2017).

**Publisher's note:** Springer Nature remains neutral with regard to jurisdictional claims in published maps and institutional affiliations.

## Supplementary Material

Supplementary Figures

## Figures and Tables

**Table 1:** The estimated incidence and mortality of liver cancer according to world area, 2012, males.

World regions	Population size, male (million)	Incidence		Mortality		Incidence: mortality ratio
		n	ASR	n	ASR	
Africa	549,445	38,693	12.4	37,012	11.8	1.05
Eastern Africa	180,243	4,556	4.9	4,337	4.6	1.07
Middle Africa	69,179	3,669	10.5	3,469	9.9	1.06
Northern Africa	106,147	13,750	18	13,109	17.4	1.03
Southern Africa	29,735	1,359	6.7	1,295	6.7	1.00
Western Africa	164,141	15,359	16.4	14,802	15.6	1.05
Asia	2,179,003	430,690	20	407,700	18.9	1.06
Eastern Asia	813,296	342,180	31.9	322,903	29.9	1.07
South-Eastern Asia	305,225	58,453	22.2	55,827	21.4	1.04
South-Central Asia	933,786	25,805	3.7	24,927	3.6	1.03
Western Asia	126,697	4,252	5	4,043	4.9	1.02
America	303,514	40,288	7.4	34,704	6.2	1.19
Caribbean	20,951	1,420	6.1	1,421	6	1.02
Central America	82,227	4,720	6.9	4,508	6.6	1.05
South America	200,336	9,980	5.2	10,417	5.4	0.96
North America	173,209	24,168	9.3	18,358	6.7	1.39
Europe	355,275	42,814	6.8	39,926	6.1	1.11
Central and Eastern Europe	138,249	9,477	4.8	10,807	5.4	0.89
Northern Europe	49,574	4,203	4.6	3,897	4	1.15
Southern Europe	74,900	14,135	9.5	12,214	7.5	1.27
Western Europe	92,553	14,999	8	13,008	6.5	1.23
Oceania	18,859	1,884	7.8	1,699	7	1.11
Australia/New Zealand	13,632	1,385	6.4	1,212	5.4	1.19
Melanesia	4,628	450	14.8	433	14.4	1.03
Micronesia/Polynesia	258	49	9.1	54	10.1	0.90
More developed regions	604,008	92,018	8.6	80,425	7.1	1.21
Less developed regions	2,975,297	462,351	17.8	440,616	17	1.05
World	3,579,305	554,369	15.3	521,041	14.3	1.07

ASR = Age standardized rate per 100,000. Source: GLOBOCAN 2012 [1]. Numbers are rounded to the nearest 10 or 100, and may not add up to the total. The population size of the world regions were retrieved from the Population Reference Bureau, Washington, DC. Available at: http://www.prb.org/Publications/Datasheets/2012/world-population-data-sheet/world-map.aspx#/table/population.

**Table 2:** The estimated incidence and mortality of liver cancer according to world area, 2012, females.

World regions	Population size, female (million)	Incidence		Mortality		Incidence: mortality ratio
		n	ASR	n	ASR	
Africa	549,608	19,987	5.8	19,045	5.6	1.04
Eastern Africa	182,469	3,391	3.3	3,193	3.1	1.06
Middle Africa	69,644	2,139	5.7	2,046	5.4	1.06
Northern Africa	105,353	5,903	7	5,595	6.7	1.04
Southern Africa	30,816	873	3.3	832	3.2	1.03
Western Africa	161,327	7,681	8.1	7,379	7.7	1.05
Asia	2,081,150	163,741	6.9	159,186	6.6	1.05
Eastern Asia	777,374	124,156	10.2	121,045	9.6	1.06
South-Eastern Asia	306,008	21,500	7.2	20,530	6.8	1.06
South-Central Asia	881,514	15,582	2.1	15,161	2.1	1.00
Western Asia	116,253	2,503	2.6	2,450	2.5	1.04
America	310,360	22,872	3.4	23,180	3.3	1.03
Caribbean	21,313	1,204	4.5	1,218	4.4	1.02
Central America	83,632	5,082	6.6	4,837	6.1	1.08
South America	205,415	8,036	3.4	8,889	3.7	0.92
North America	176,585	8,550	2.7	8,236	2.3	1.17
Europe	381,747	20,648	2.2	22,265	2.2	1.00
Central and Eastern Europe	155,701	6,476	2	7,611	2.2	0.91
Northern Europe	51,252	2,254	1.8	2,418	1.8	1.00
Southern Europe	78,393	6,423	2.9	6,374	2.5	1.16
Western Europe	96,400	5,495	2.2	5,862	2.1	1.05
Oceania	18,746	834	3.1	816	2.9	1.07
Australia/New Zealand	13,715	569	2.1	562	2	1.05
Melanesia	4,451	256	7.6	241	7.3	1.04
Micronesia/Polynesia	580	9	1.4	13	2.2	0.64
More developed regions	637,294	42,284	2.7	42,652	2.5	1.08
Less developed regions	2,880,901	185,798	6.6	181,840	6.4	1.03
World	3,518,195	228,082	5.4	224,492	5.1	1.06

ASR = Age standardized rate per 100,000. Source: GLOBOCAN 2012 [1]. Numbers are rounded to the nearest 10 or 100, and may not add up to the total. The population size of the world regions were retrieved from the Population Reference Bureau, Washington, DC. Available at: http://www.prb.org/Publications/Datasheets/2012/world-population-data-sheet/world-map.aspx#/table/population.
