# Supplementary material for: International incidence and mortality trends of liver cancer: a global profile
Source: Sci Rep. 2017 Mar 31;7:45846. doi: 10.1038/srep45846 (PMC5374459; doi:10.1038/srep45846)
Supplement: Supplementary Figures [file srep45846-s1.pdf]

International incidence and mortality trends of liver cancer: a global profile

Martin CS Wong MD, MPH[1, 2,*], Johnny JY Jiang MD, PhD[3], William B Goggins PhD (Harvard)[1], Miaoyin Liang MPH[1], Yuan Fang PhD[1], Franklin D Fung BHSc (Hons)[1], Colette Leung BSc (Hons)[1], Harry HX Wang PhD[4, 5], Grace LH Wong MD, FRCP[2, 6, 7], Vincent WS Wong MD, FRCP[2, 6, 7], Henry LY Chan MD, FRCP[2, 6, 7]

1. School of Public Health and Primary Care, Faculty of Medicine, Chinese University of Hong Kong. Prince of Wales Hospital, Shatin, New Territories, Hong Kong
2. Institute of Digestive Disease, Faculty of Medicine, Chinese University of Hong Kong
3. Peking Union School of Public Health, Chinese Academy of Medical Sciences and Peking Union Medical College, China
4. School of Public Health, Sun Yat-Sen University, Guangzhou, 510080, P.R. China;
5. General Practice and Primary Care, Institute of Health and Wellbeing, University of Glasgow, Glasgow G12 9LX, UK;
6. Department of Medicine and Therapeutics, Faculty of Medicine, Chinese University of Hong Kong
7. State Key Laboratory of Digestive Disease, Faculty of Medicine, Chinese University of Hong Kong

Correspondence on submission: Martin CS Wong BMedSc (Hons), MSc (Hons), MBChB, MD, MPH, MBA, FRSPH, FRSP, FRIPH, FRACGP, FHKCFP, DCH, FHKAM, FHKAN
Corresponding Author: Henry LY Chan MBChB (CUHK), MD (CUHK), FRCP (Edin), FRCP (Lond), FHKCP, FHKAM (Med)

Professor, Department of Medicine and Therapeutics; Head, Division of Gastroenterology and Hepatology; Director, Institute of Digestive Disease; Director, Center for Liver Health, Faculty of Medicine, The Chinese University of Hong Kong

Phone: 852-26321506; **Fax**: 852-26373852; **Email**: hlychan@cuhk.edu.hk

Supplementary Figure 1 Temporal trends in the incidence and mortality of liver cancer according to country and gender*

1) Latin America and the Caribbean

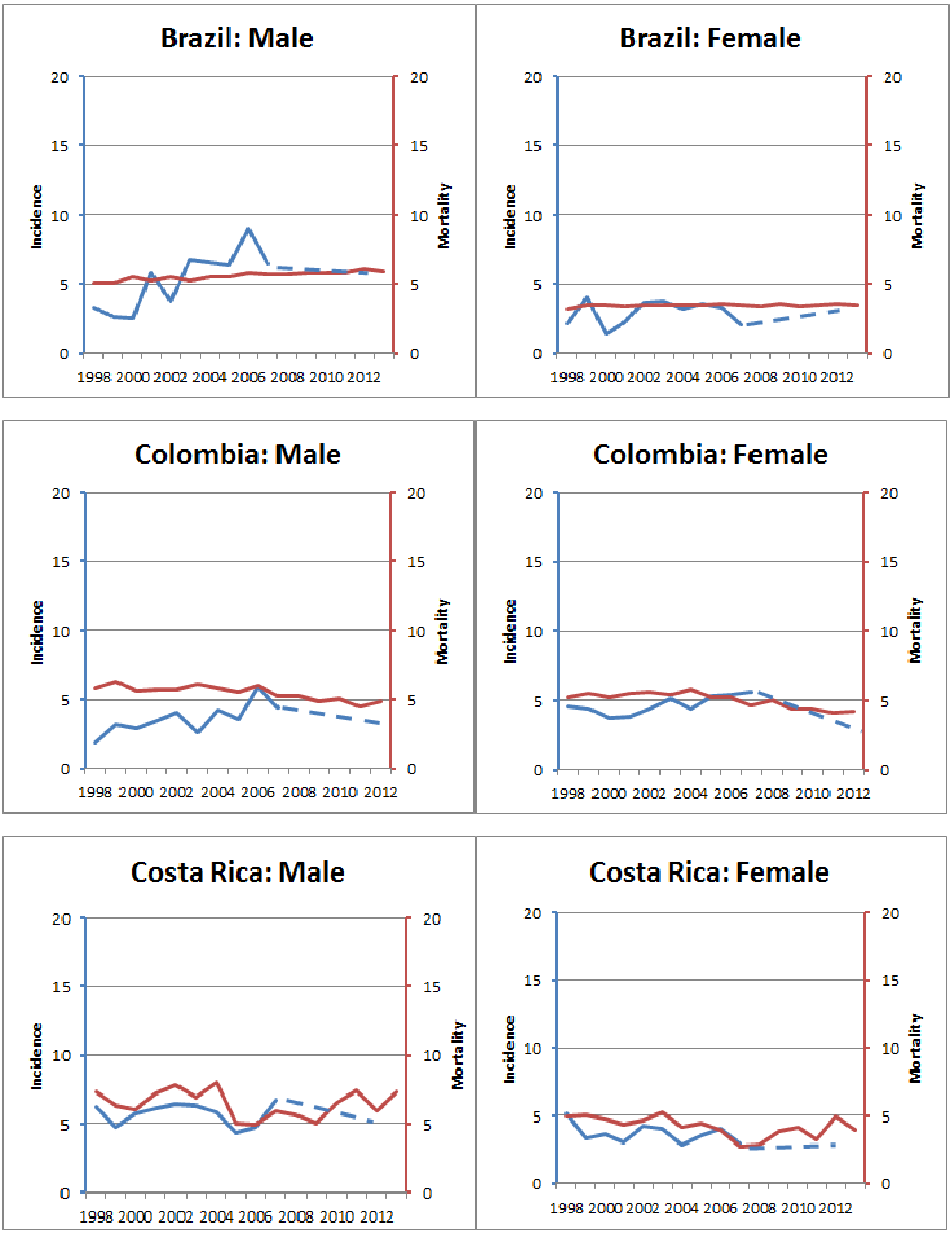

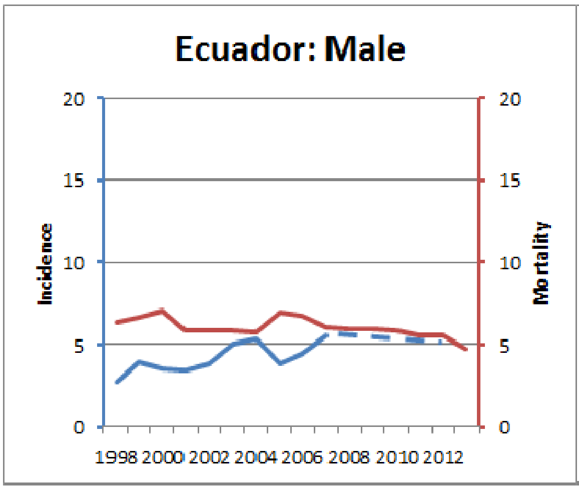

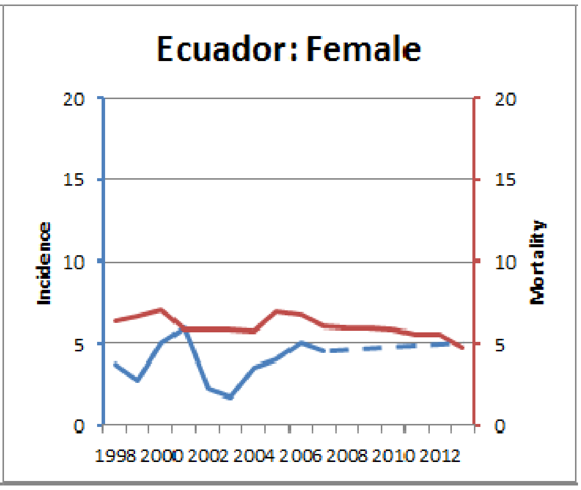

2) Northern America

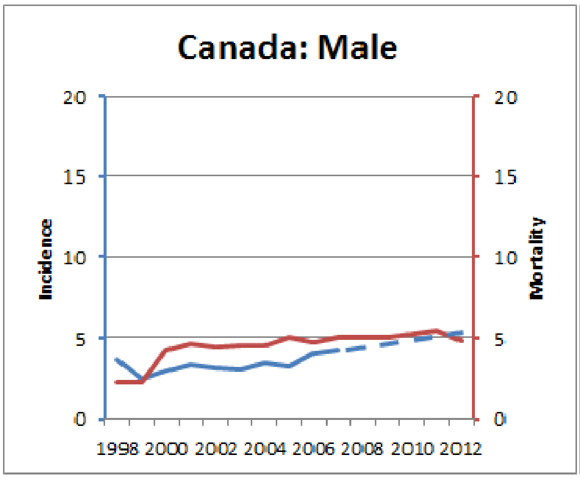

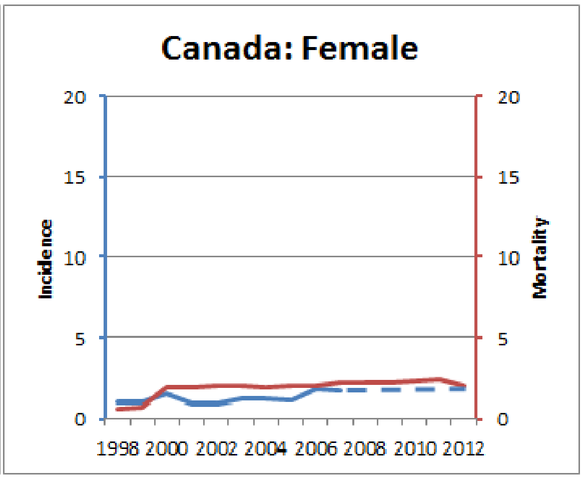

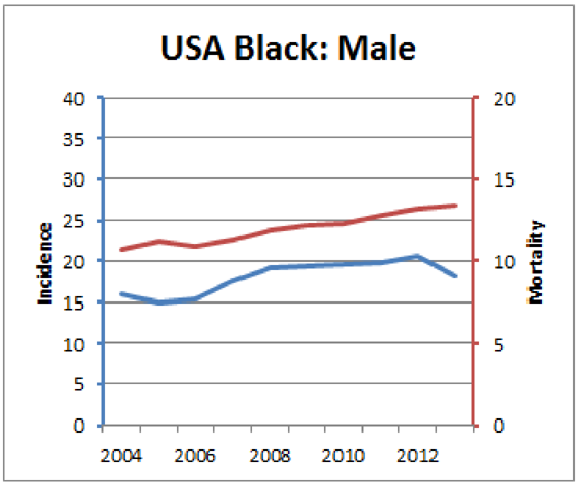

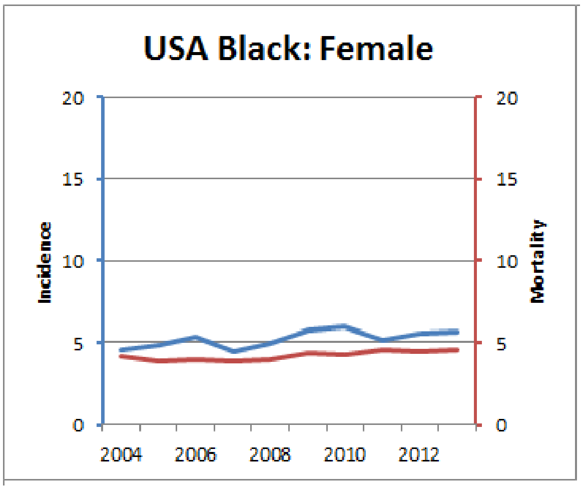

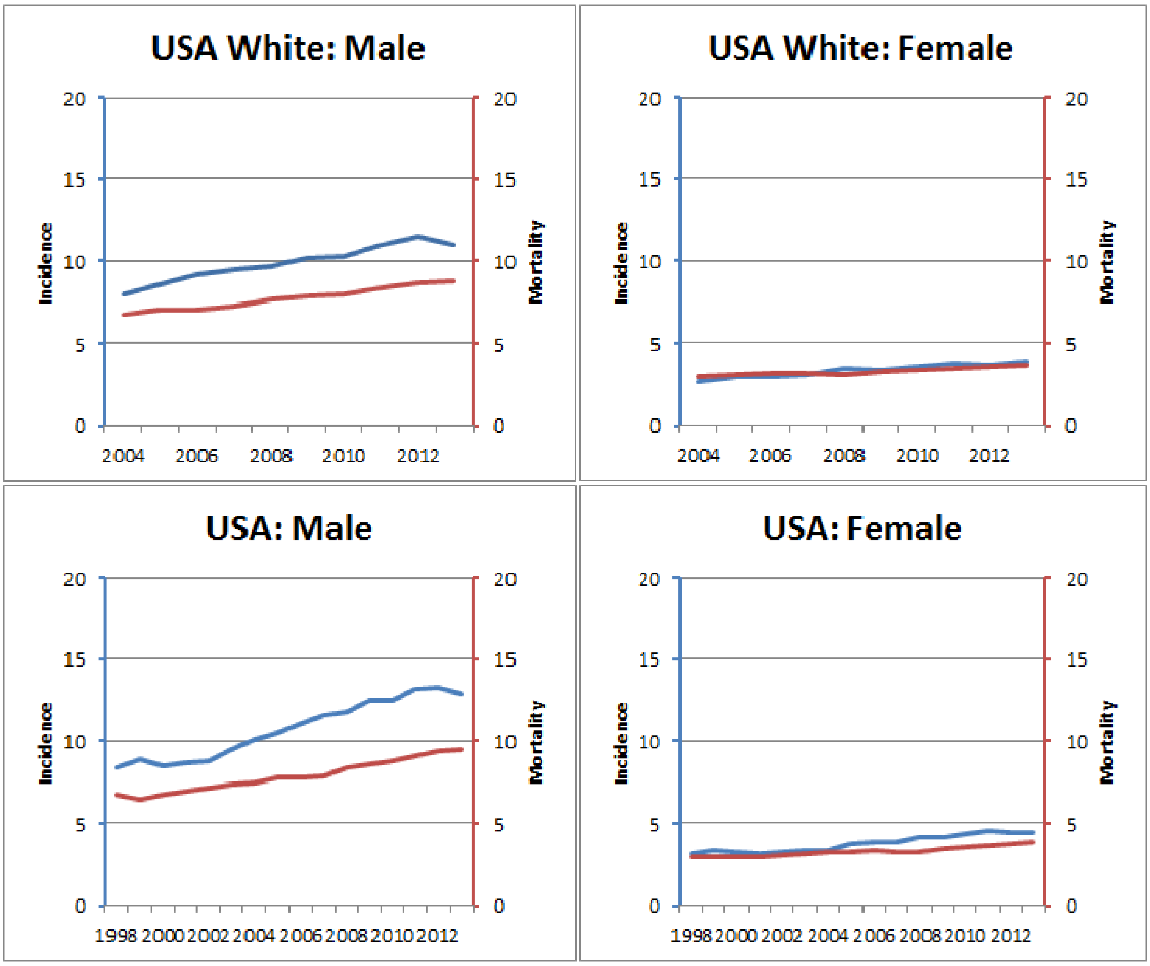

USA White: Male
USA White: Female
USA: Male
USA: Female
Incidence
Mortality

3) Asia

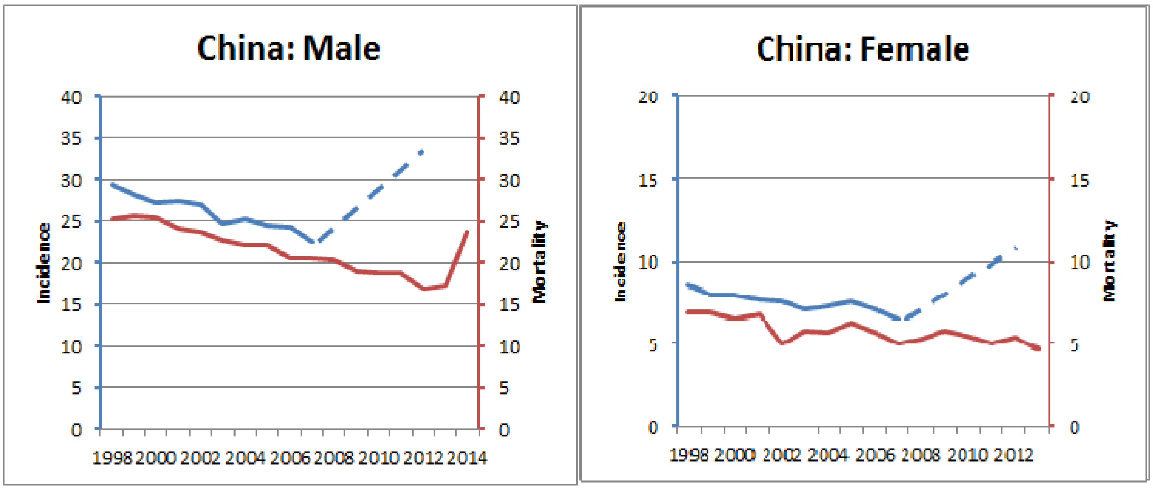

China: Male
China: Female
Incidence
Mortality

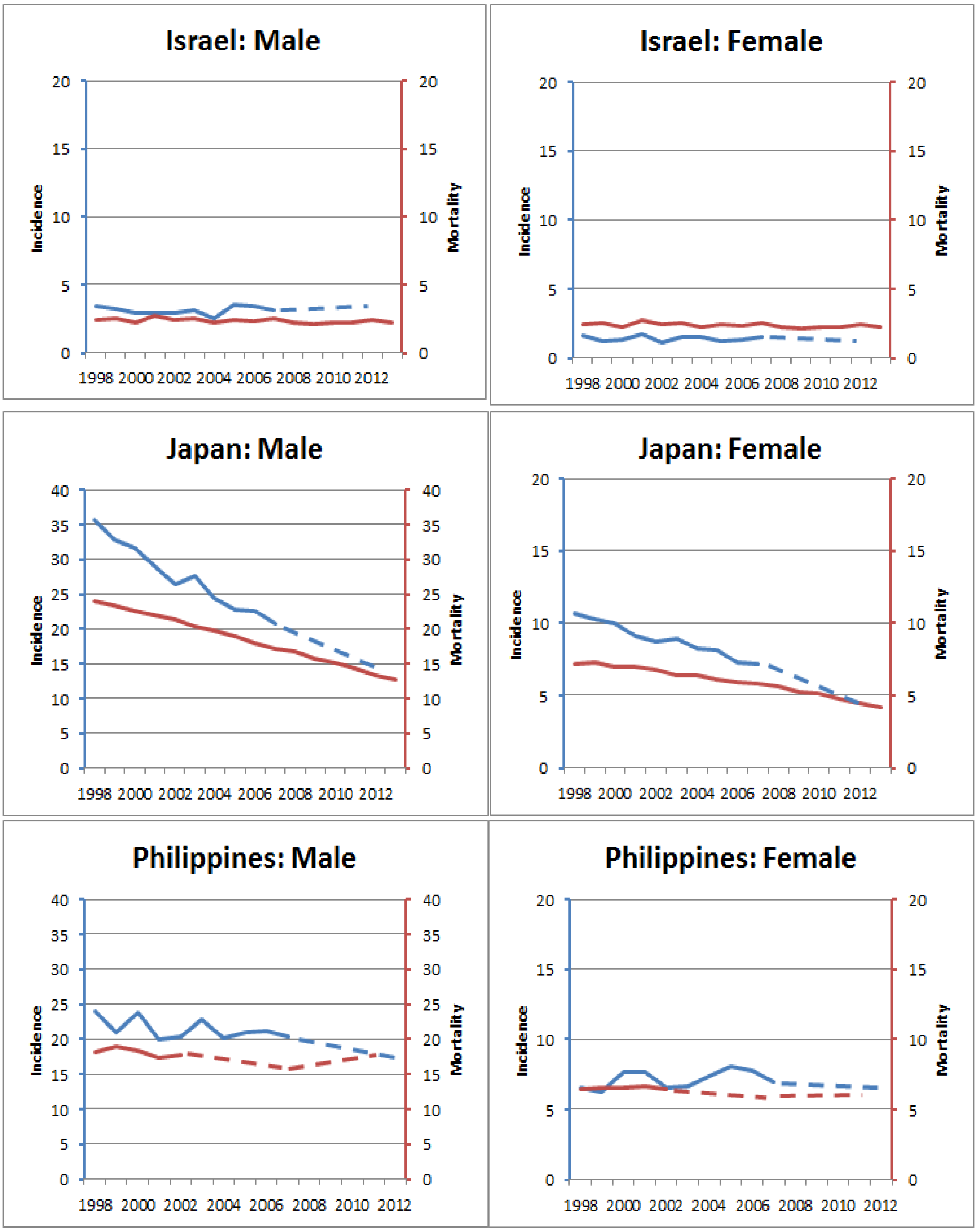
Israel: Male
Israel: Female
Japan: Male
Japan: Female
Philippines: Male
Philippines: Female
Incidence
Mortality
1998 2000 2002 2004 2006 2008 2010 2012

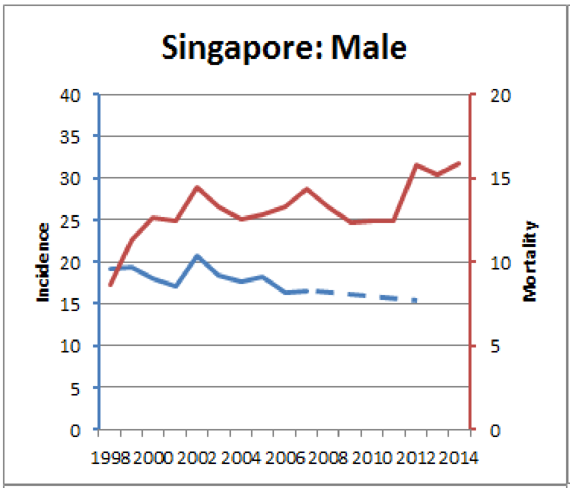
Singapore: Male
Incidence
Mortality
40 35 30 25 20 15 10 5 0
20 15 10 5 0
1998 2000 2002 2004 2006 2008 2010 2012 2014

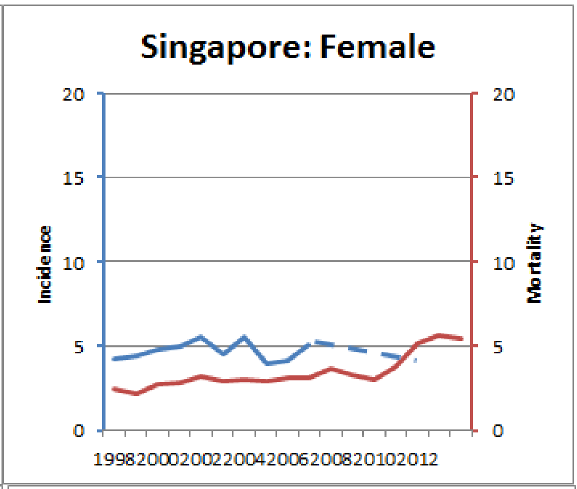
Singapore: Female
Incidence
Mortality
20 15 10 5 0
20 15 10 5 0
1998 2000 2002 2004 2006 2008 2010 2012

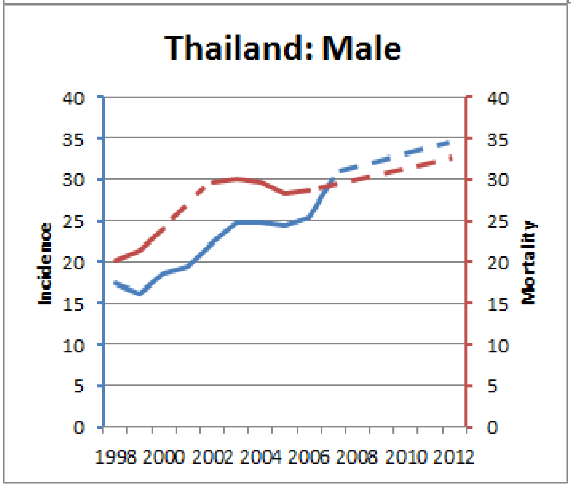
Thailand: Male
Incidence
Mortality
40 35 30 25 20 15 10 5 0
40 35 30 25 20 15 10 5 0
1998 2000 2002 2004 2006 2008 2010 2012

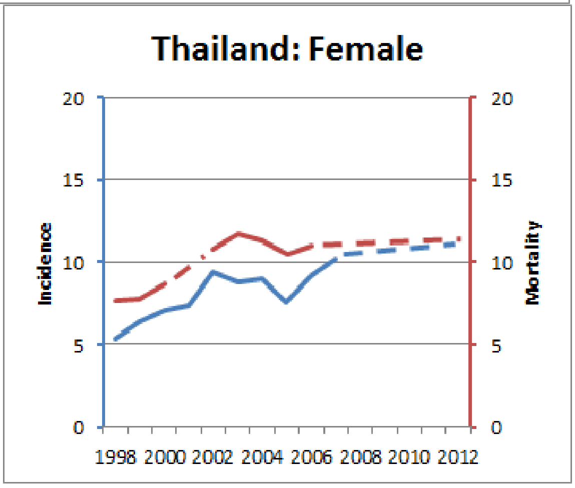
Thailand: Female
Incidence
Mortality
20 15 10 5 0
20 15 10 5 0
1998 2000 2002 2004 2006 2008 2010 2012

4) Oceania

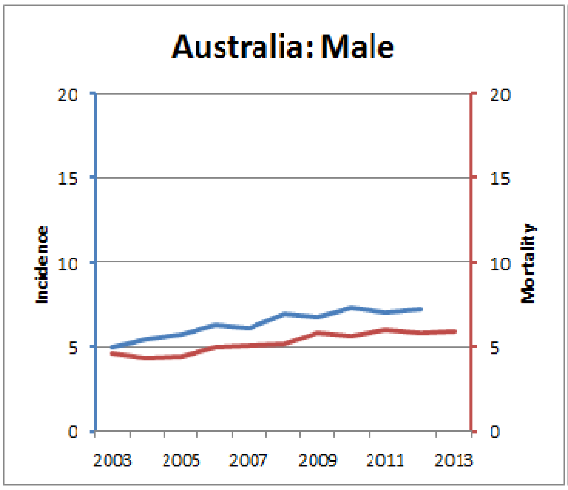
Australia: Male
Incidence
Mortality
20 15 10 5 0
20 15 10 5 0
2003 2005 2007 2009 2011 2013

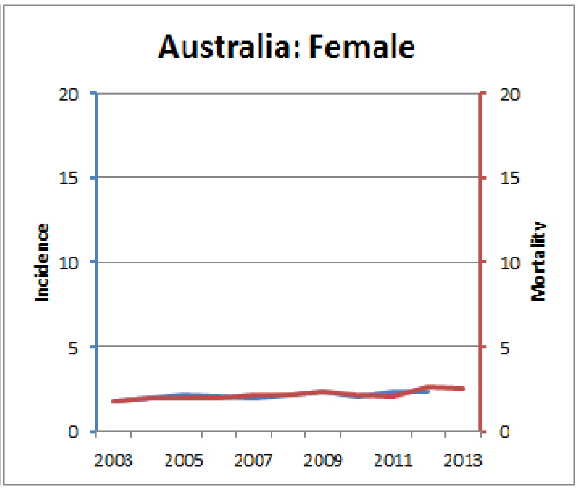
Australia: Female
Incidence
Mortality
20 15 10 5 0
20 15 10 5 0
2003 2005 2007 2009 2011 2013

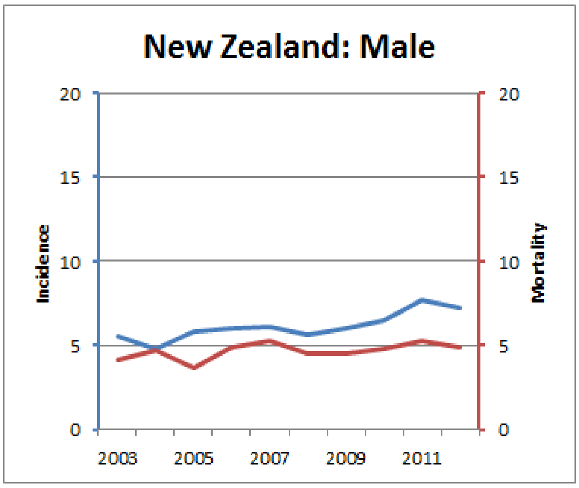

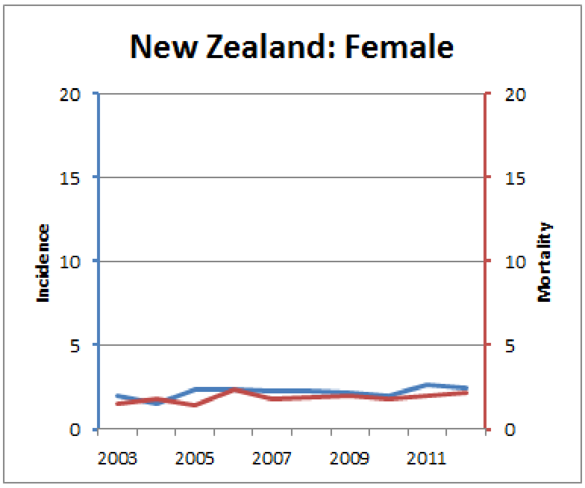

5) Northern Europe

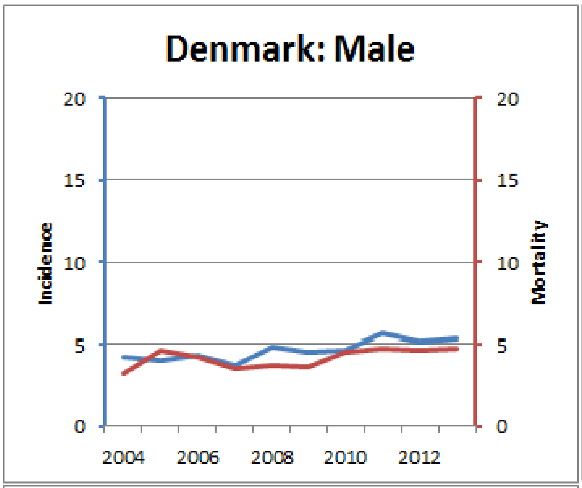

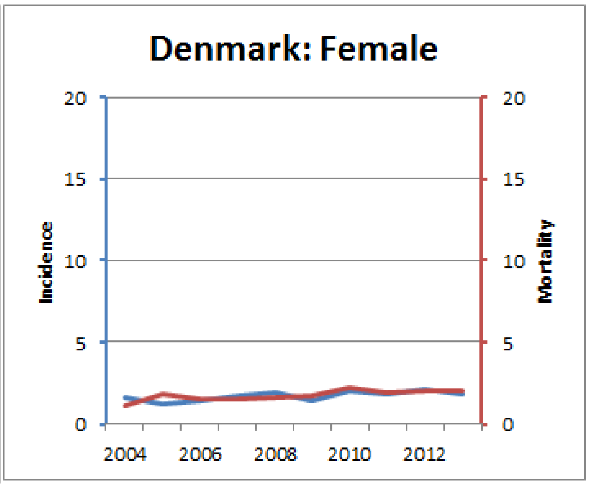

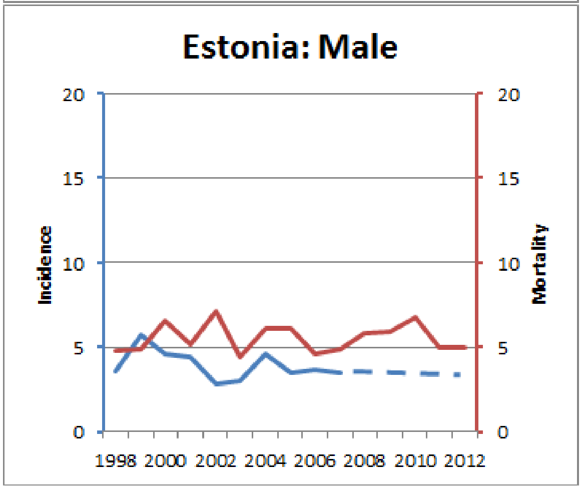

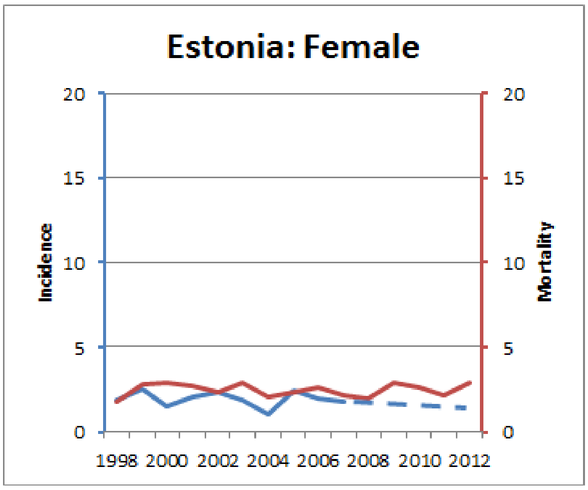

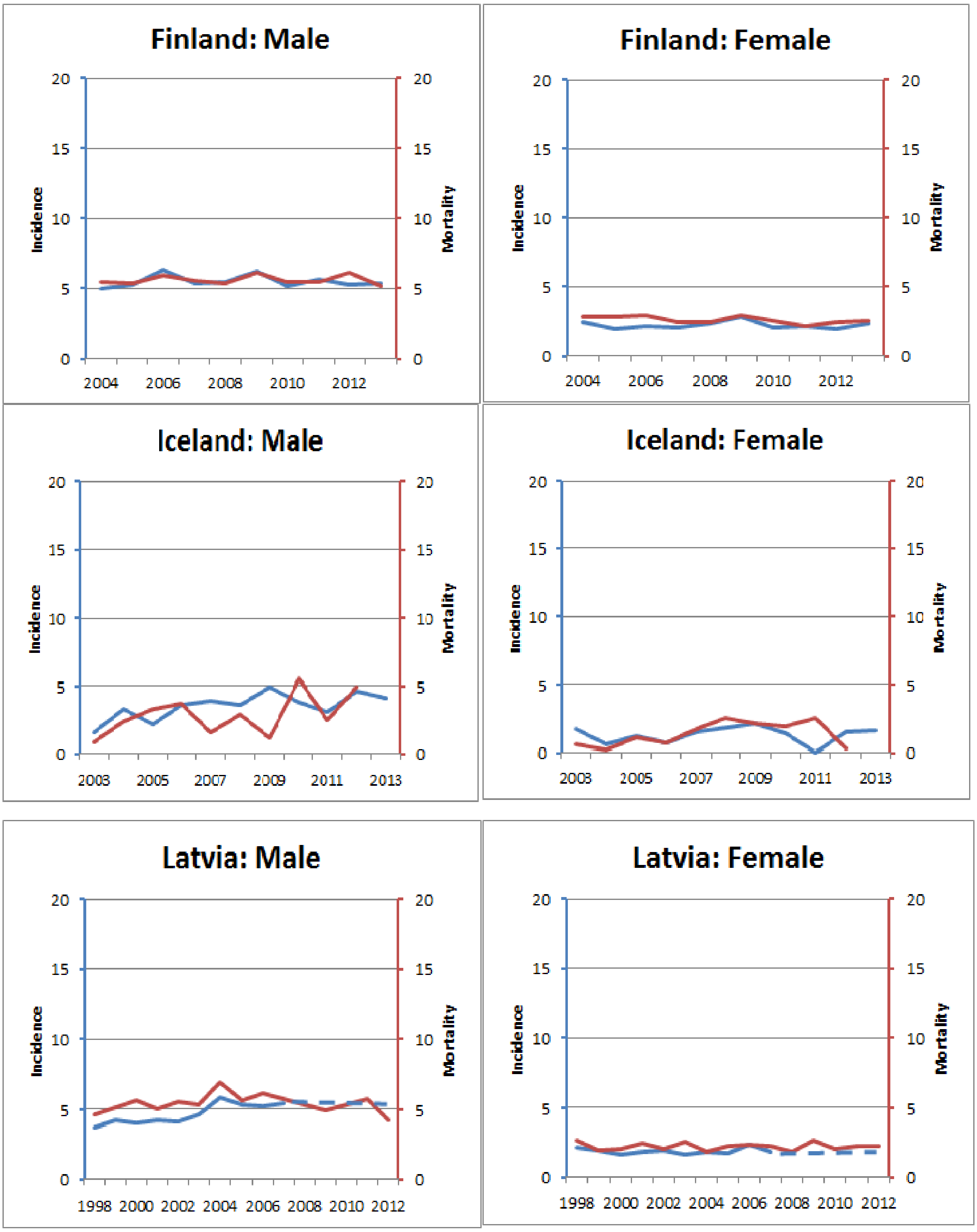
Finland: Male
Incidence
Mortality
0 5 10 15 20
2004 2006 2008 2010 2012
Finland: Female
Incidence
Mortality
0 5 10 15 20
2004 2006 2008 2010 2012
Iceland: Male
Incidence
Mortality
0 5 10 15 20
2003 2005 2007 2009 2011 2013
Iceland: Female
Incidence
Mortality
0 5 10 15 20
2003 2005 2007 2009 2011 2013
Latvia: Male
Incidence
Mortality
0 5 10 15 20
1998 2000 2002 2004 2006 2008 2010 2012
Latvia: Female
Incidence
Mortality
0 5 10 15 20
1998 2000 2002 2004 2006 2008 2010 2012

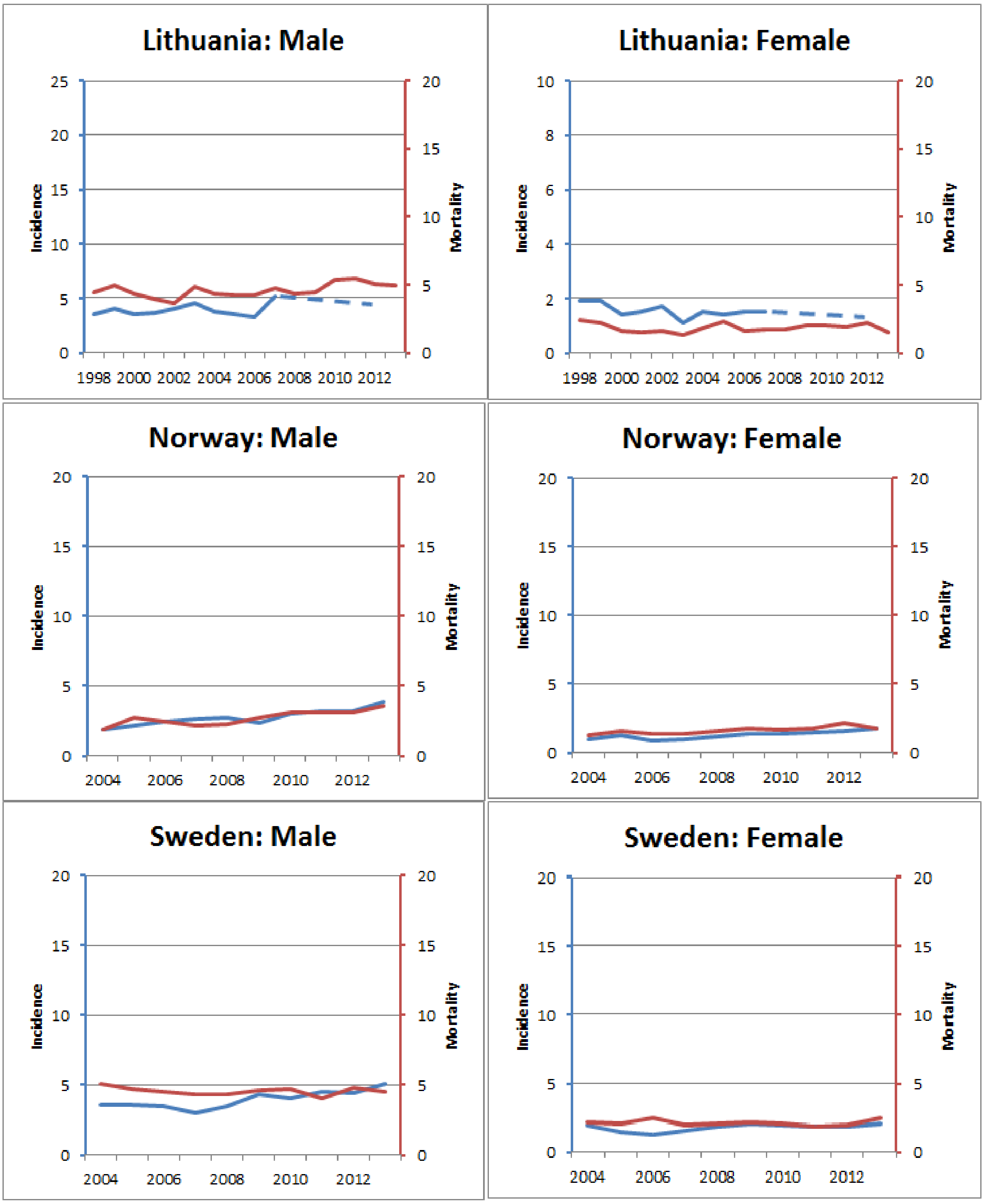
Lithuania: Male
Incidence
Mortality
25 20 15 10 5 0
20 15 10 5 0
1998 2000 2002 2004 2006 2008 2010 2012
Lithuania: Female
Incidence
Mortality
10 8 6 4 2 0
20 15 10 5 0
1998 2000 2002 2004 2006 2008 2010 2012
Norway: Male
Incidence
Mortality
20 15 10 5 0
20 15 10 5 0
2004 2006 2008 2010 2012
Norway: Female
Incidence
Mortality
20 15 10 5 0
20 15 10 5 0
2004 2006 2008 2010 2012
Sweden: Male
Incidence
Mortality
20 15 10 5 0
20 15 10 5 0
2004 2006 2008 2010 2012
Sweden: Female
Incidence
Mortality
20 15 10 5 0
20 15 10 5 0
2004 2006 2008 2010 2012

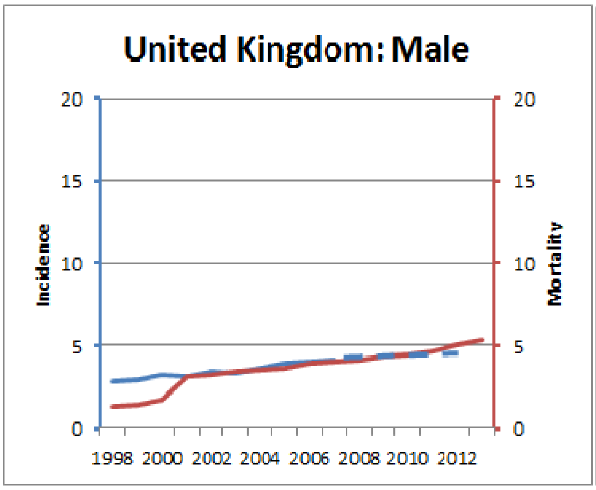

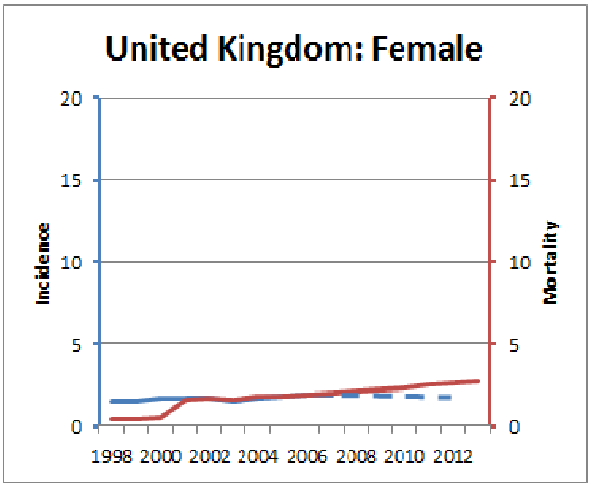

6) Western Europe

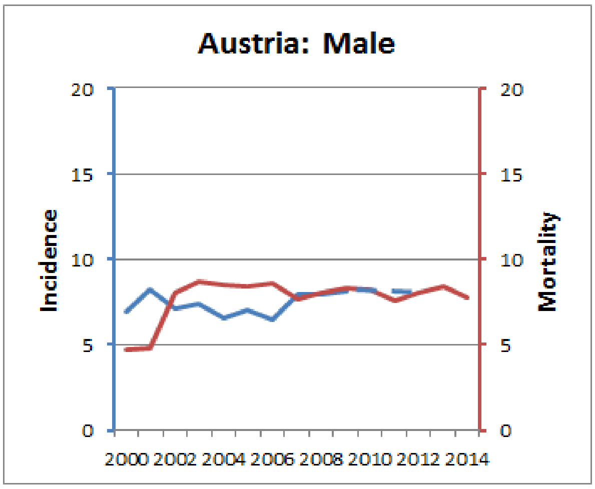

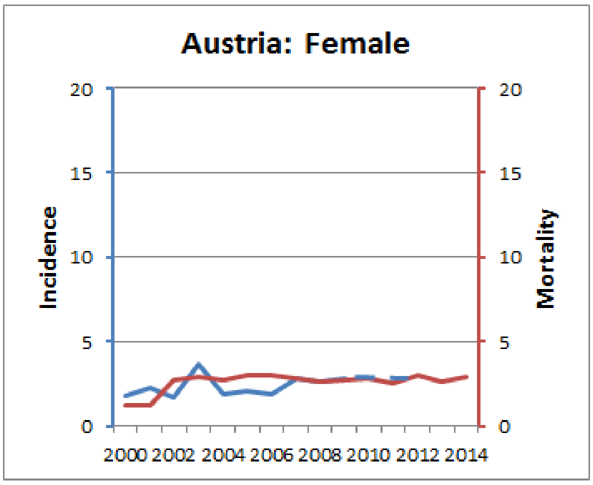

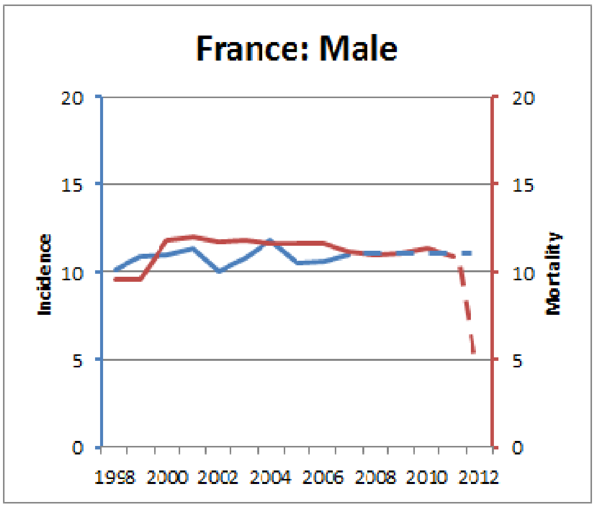

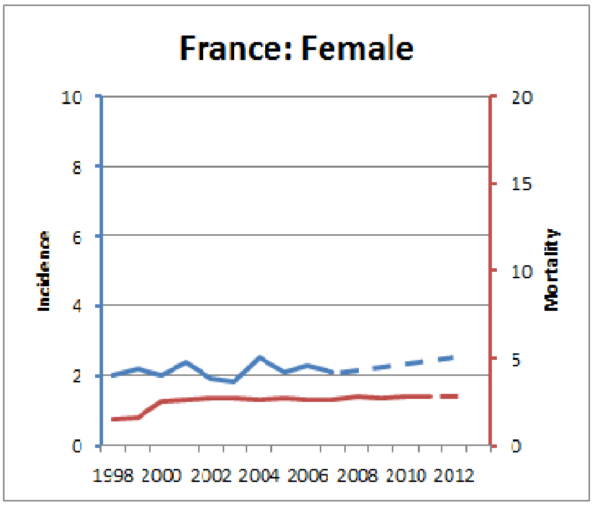

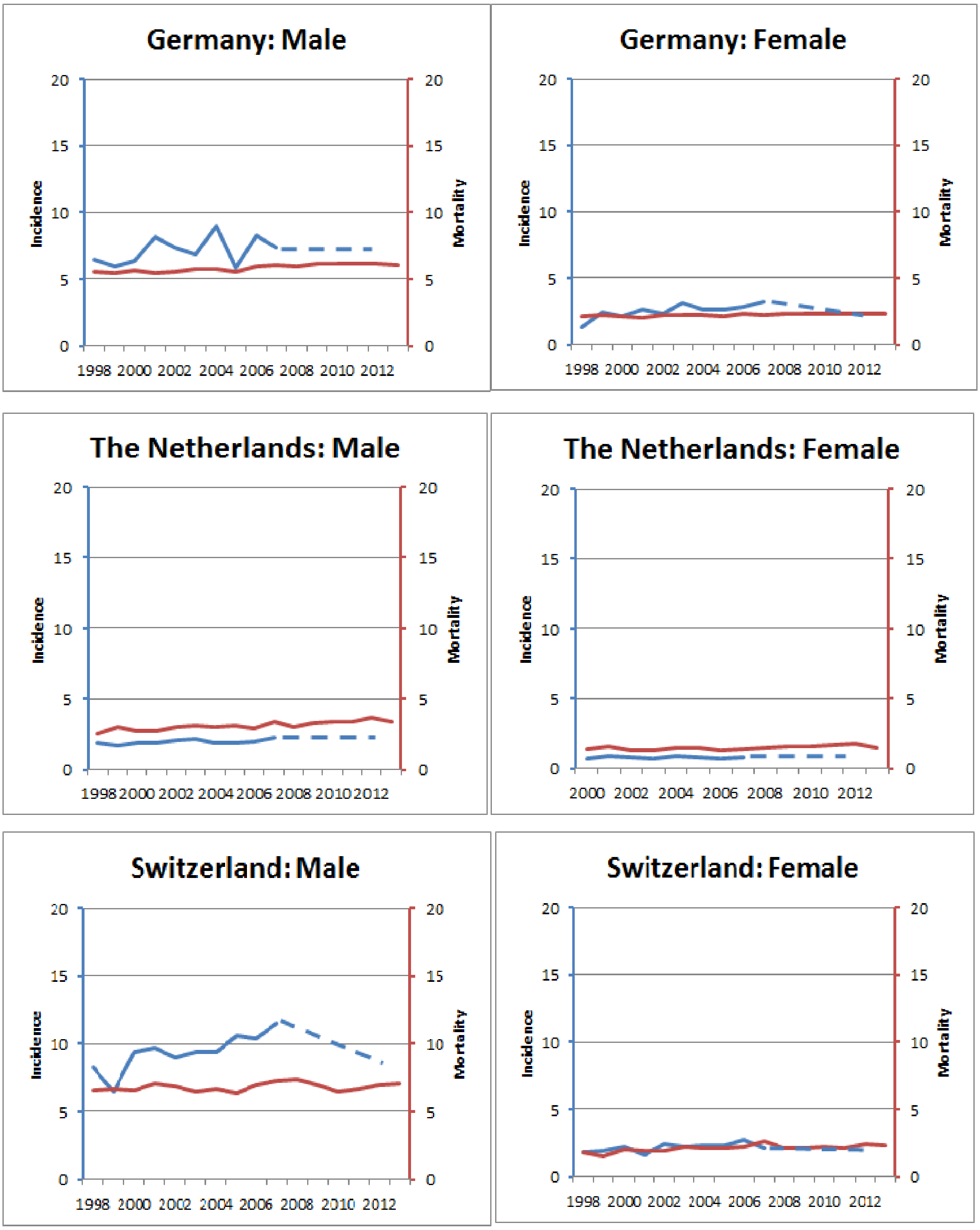
Germany: Male
Germany: Female
The Netherlands: Male
The Netherlands: Female
Switzerland: Male
Switzerland: Female
Incidence
Mortality
0
5
10
15
20
1998 2000 2002 2004 2006 2008 2010 2012
2000 2002 2004 2006 2008 2010 2012

7) Southern Europe

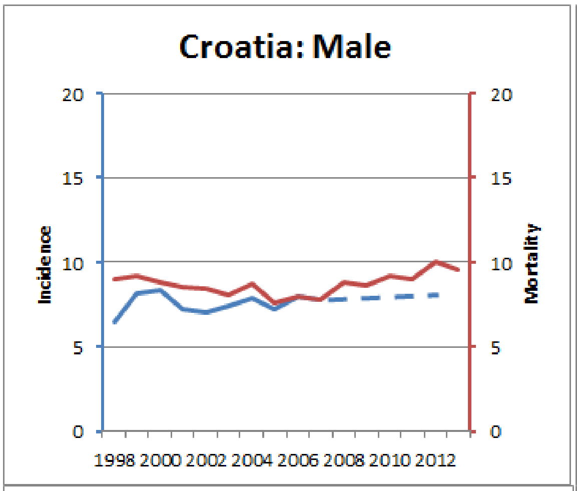

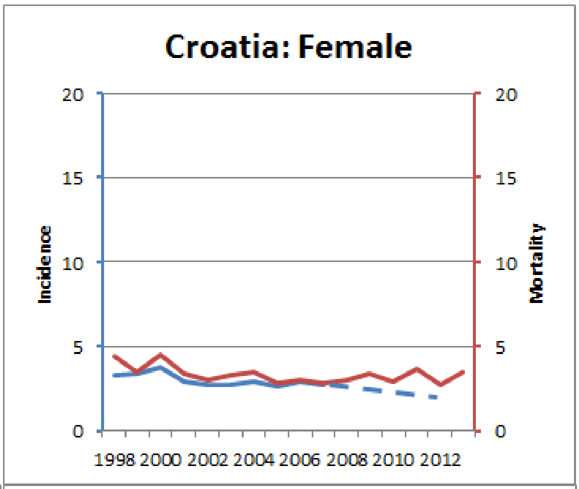

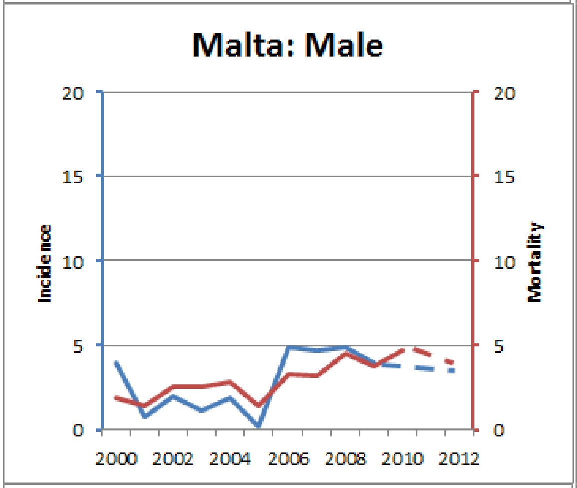

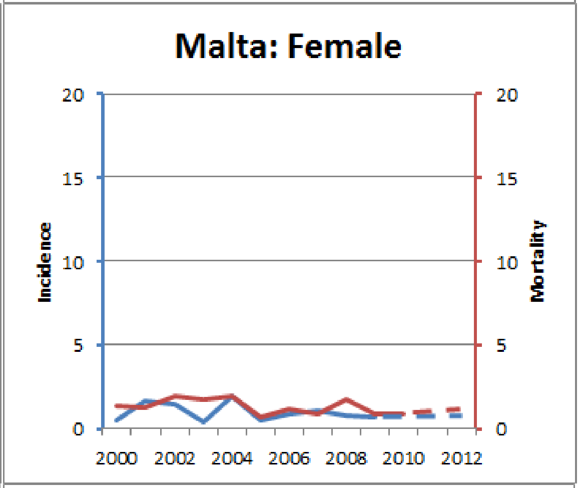

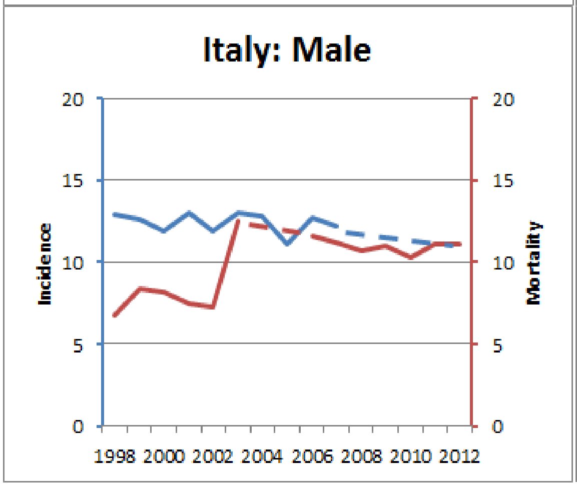

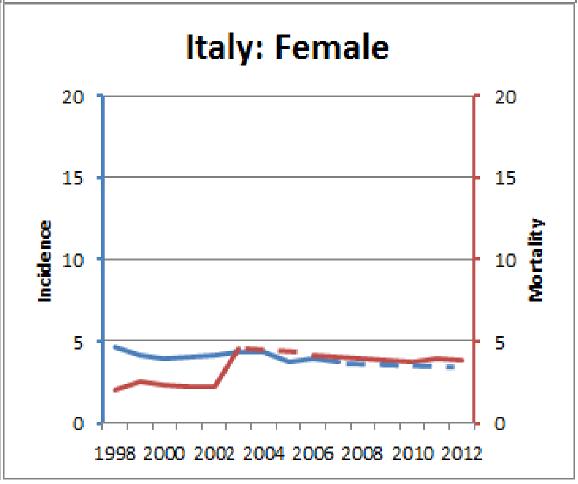

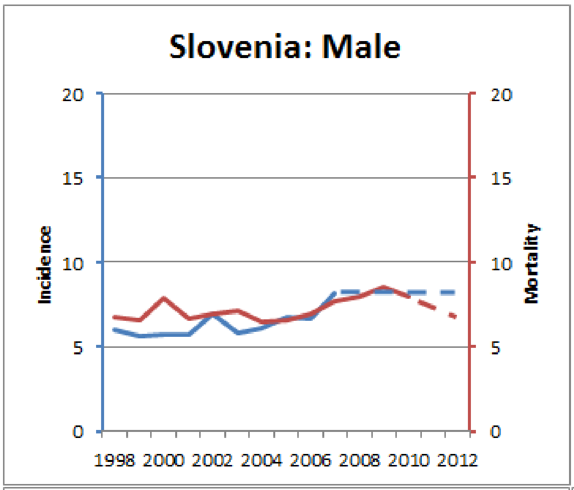

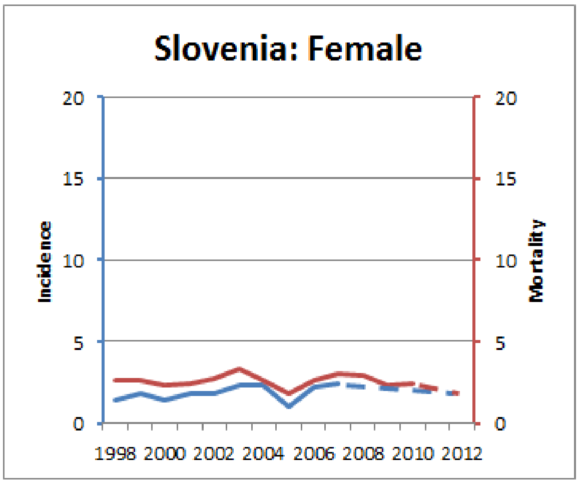

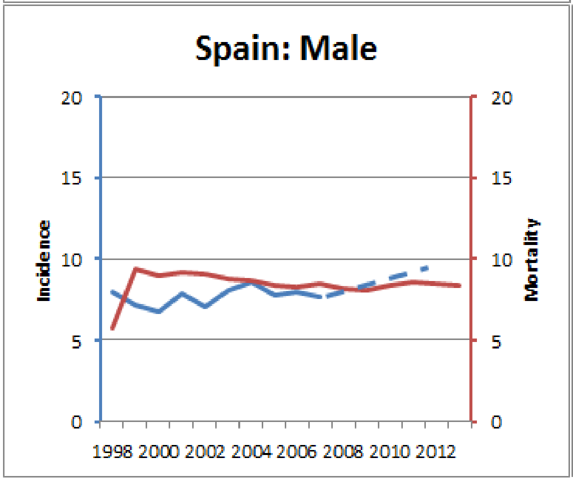

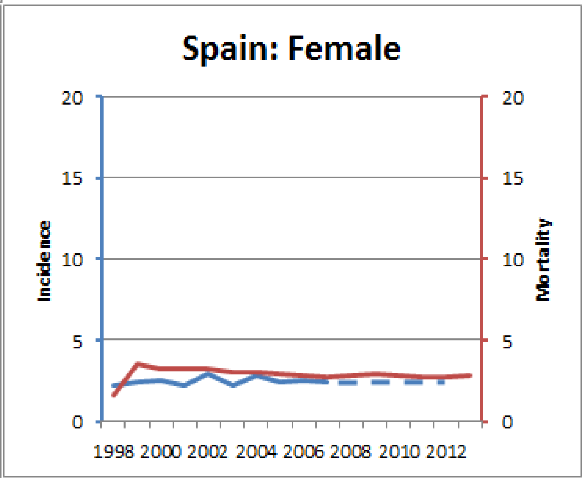

8) Eastern Europe

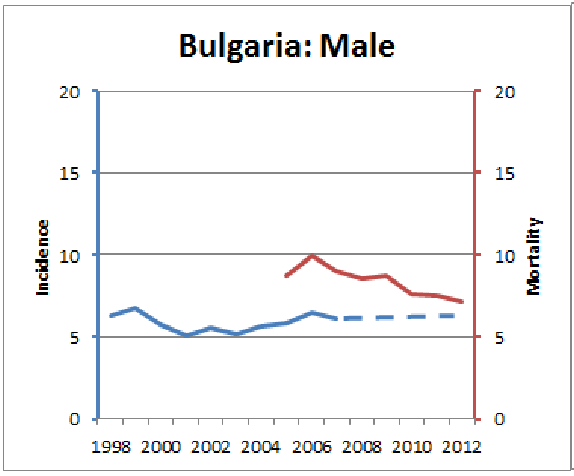

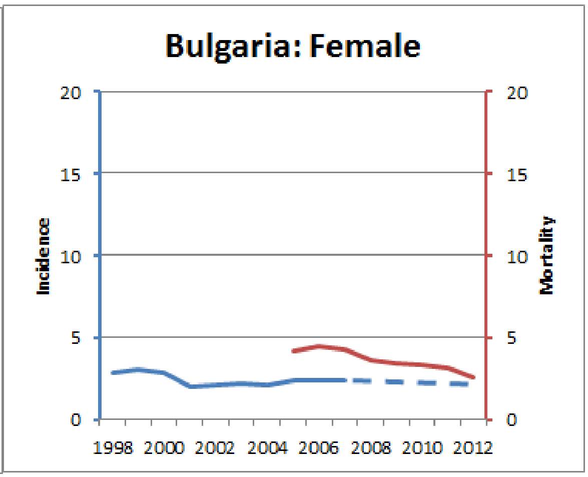

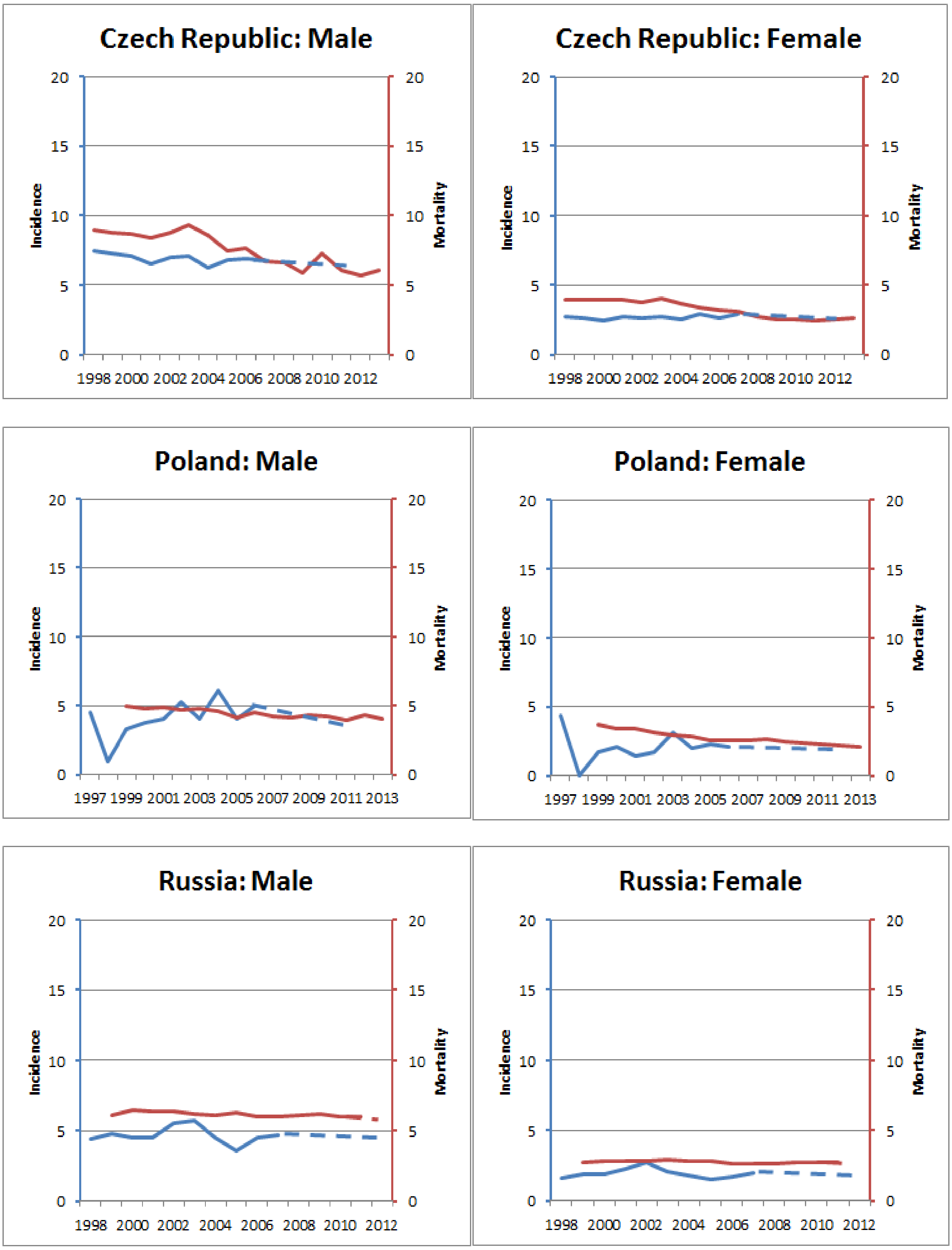
Czech Republic: Male
Czech Republic: Female
Poland: Male
Poland: Female
Russia: Male
Russia: Female
Incidence
Mortality
0
5
10
15
20
1998 2000 2002 2004 2006 2008 2010 2012
1997 1999 2001 2003 2005 2007 2009 2011 2013

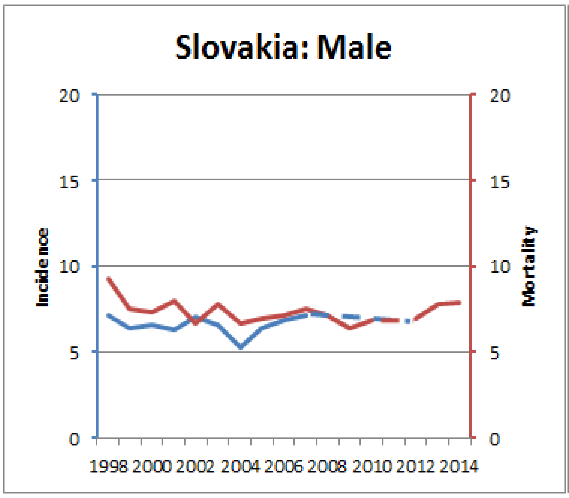
Slovakia: Male
Incidence
Mortality
20
15
10
5
0
1998 2000 2002 2004 2006 2008 2010 2012 2014

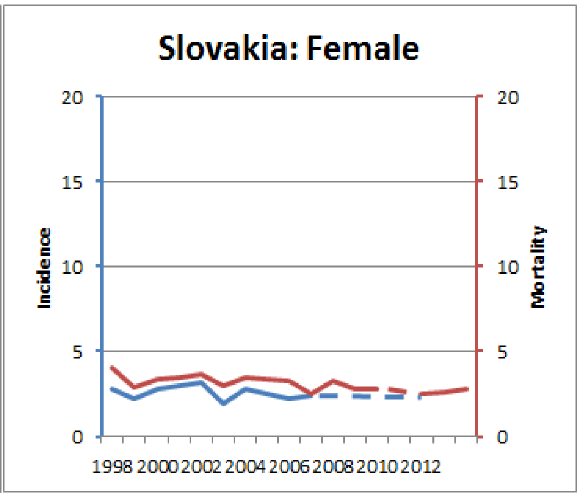
Slovakia: Female
Incidence
Mortality
20
15
10
5
0
1998 2000 2002 2004 2006 2008 2010 2012

Supplementary Figure 2 Findings from the joinpoint regression analysis of the global incidence rates of liver cancer (Left: Male, Right: Female)

Latin America and the Caribbean

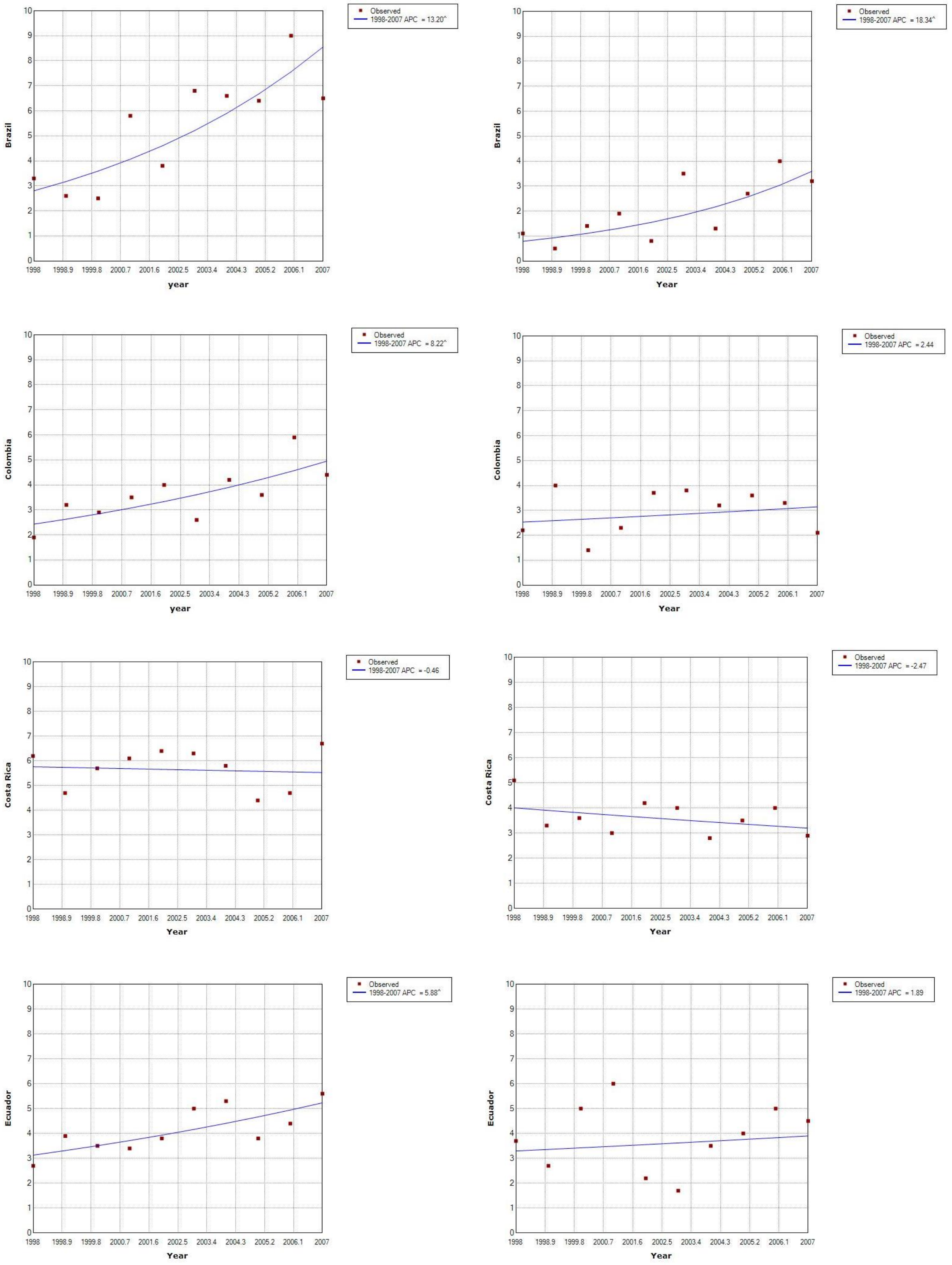

1) Northern America

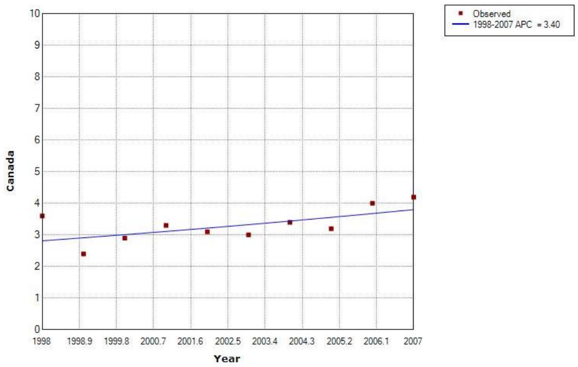

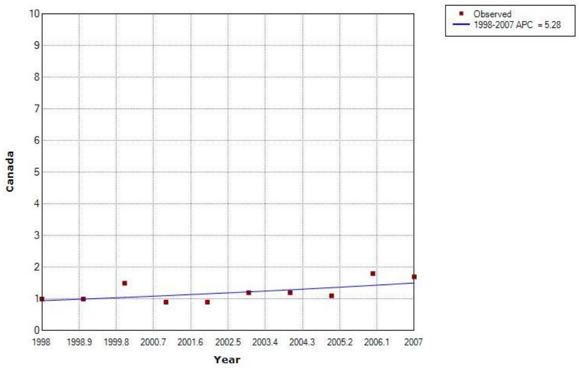

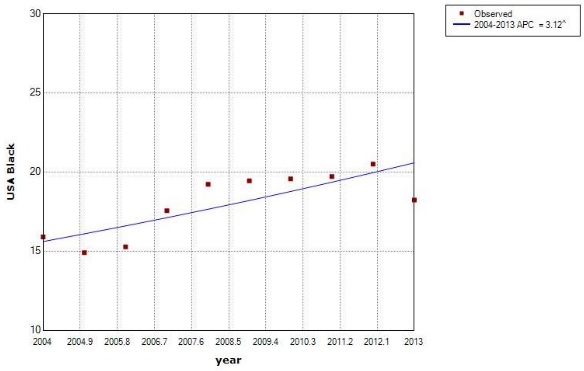

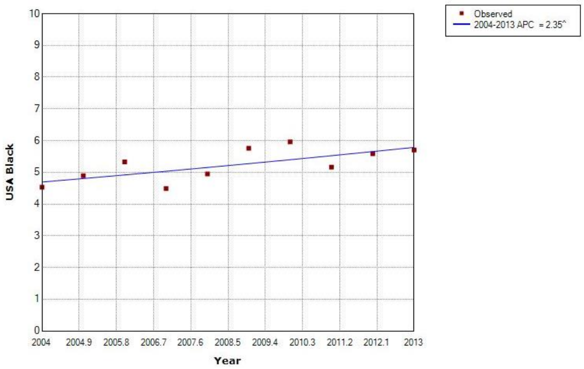

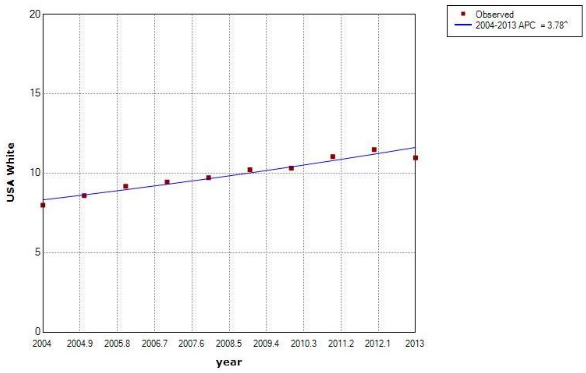

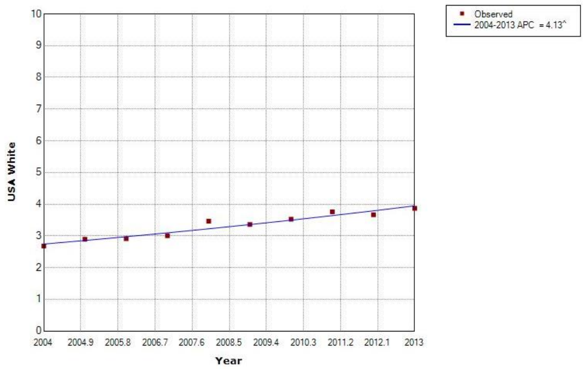

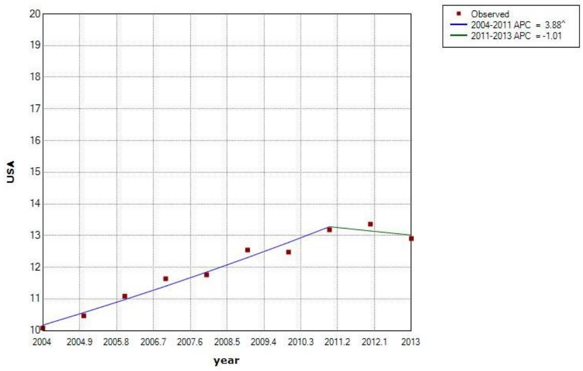

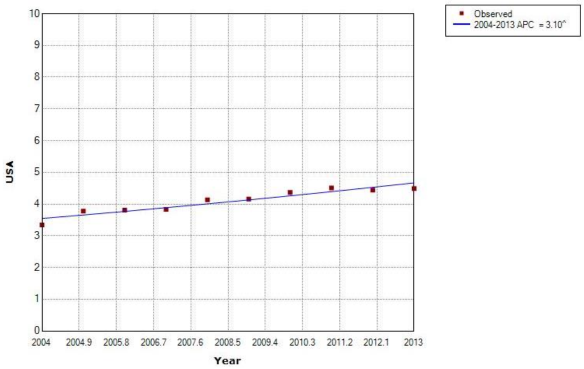

2) Asia

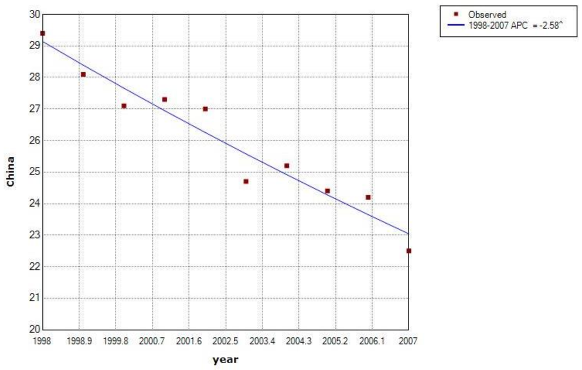
Observed
1998-2007 APC = -2.58^
China
year

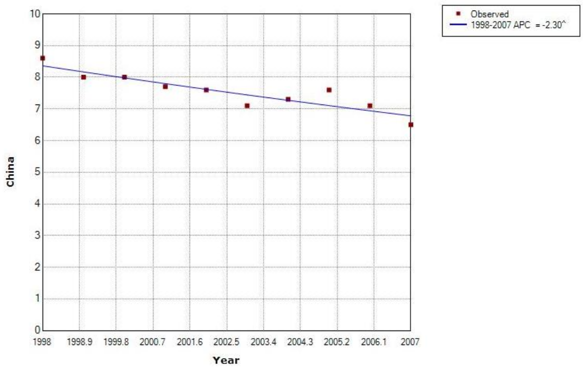
Observed
1998-2007 APC = -2.30^
China
Year

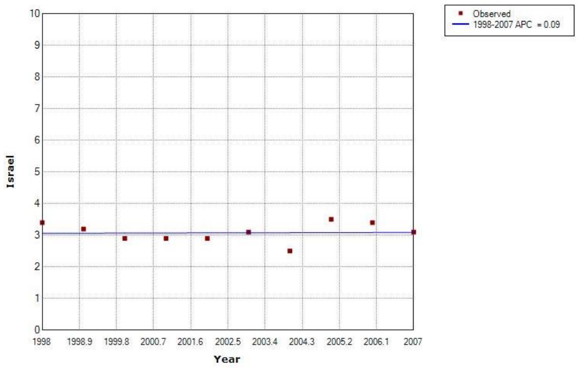
Observed
1998-2007 APC = 0.09
Israel
Year

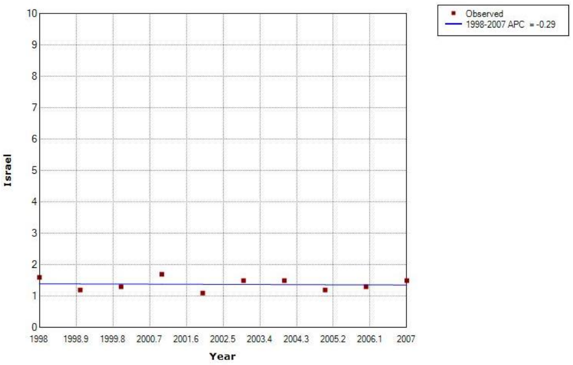
Observed
1998-2007 APC = -0.29
Israel
Year

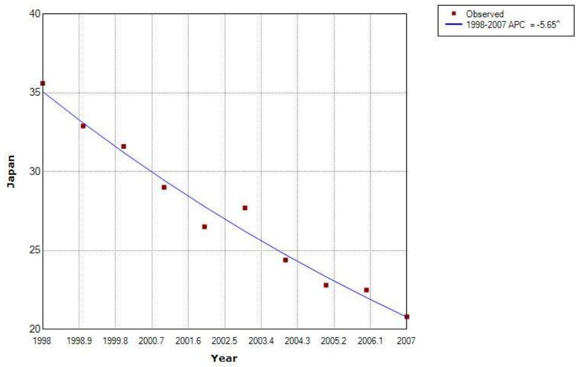
Observed
1998-2007 APC = -5.65^
Japan
Year

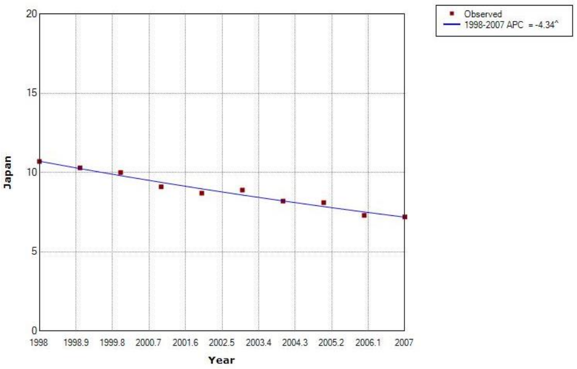
Observed
1998-2007 APC = -4.34^
Japan
Year

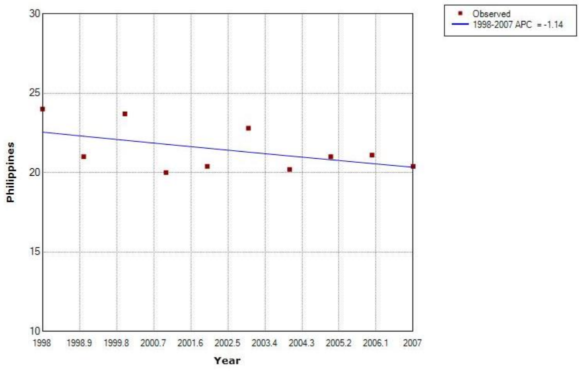
Observed
1998-2007 APC = -1.14
Philippines
Year

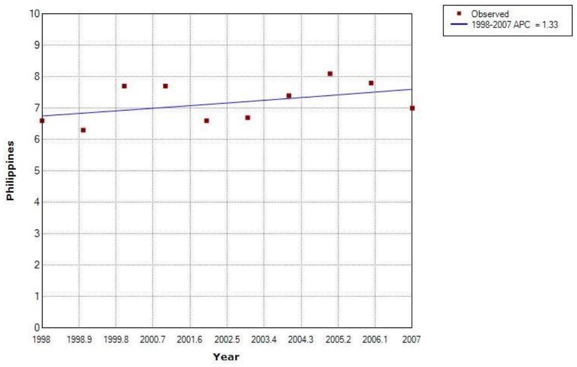
Observed
1998-2007 APC = 1.33
Philippines
Year

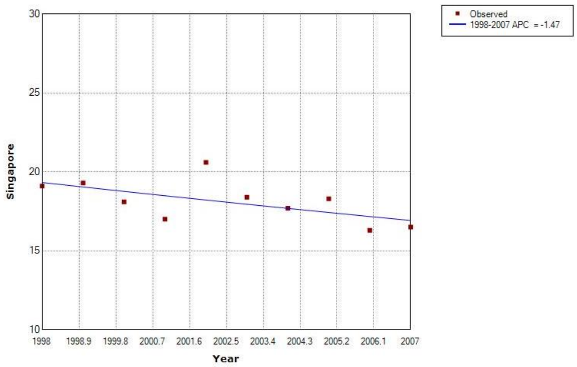

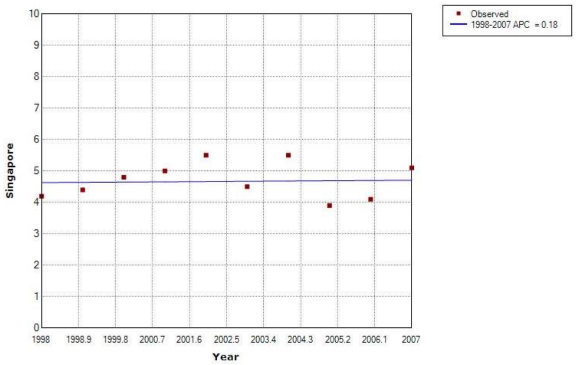

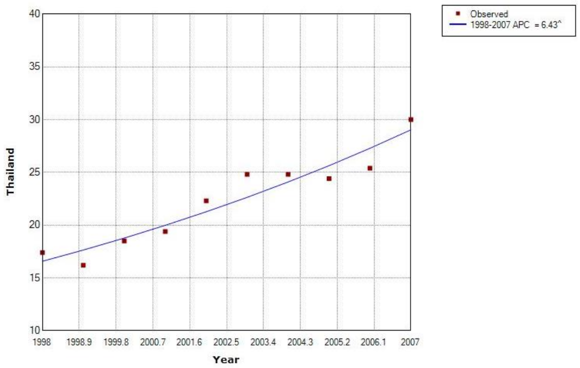

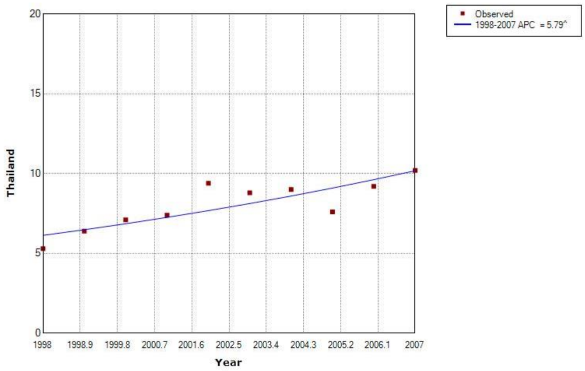

3) Oceania

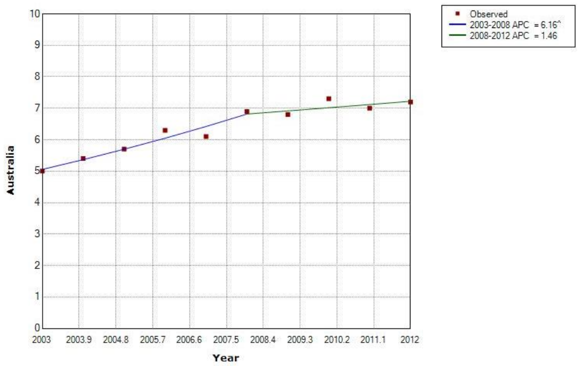

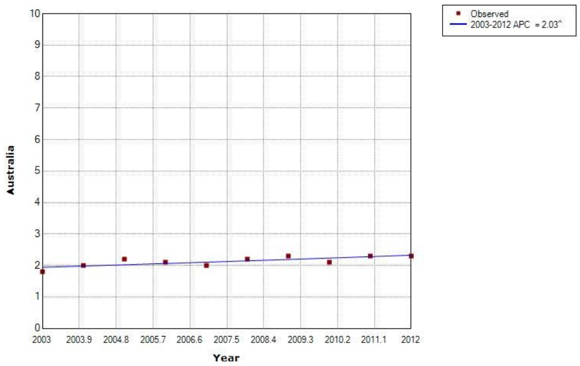

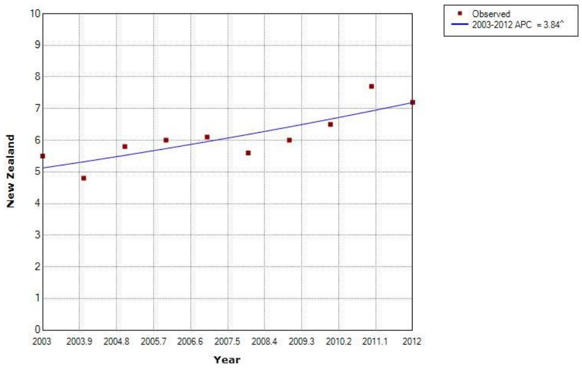

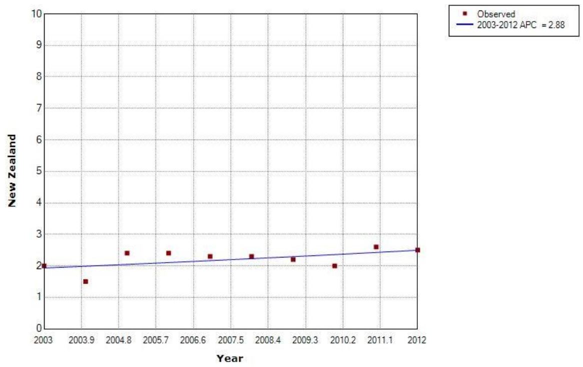

4) Northern Europe

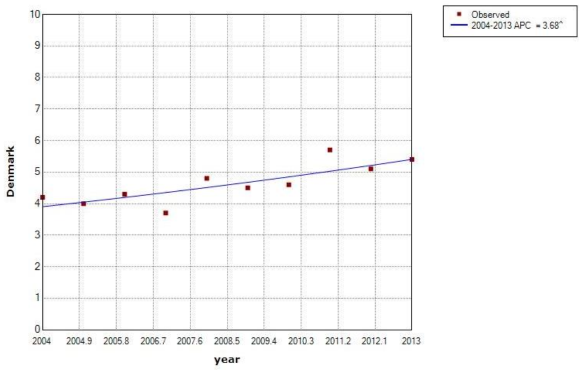
Observed
2004-2013 APC = 3.68^
Denmark
year

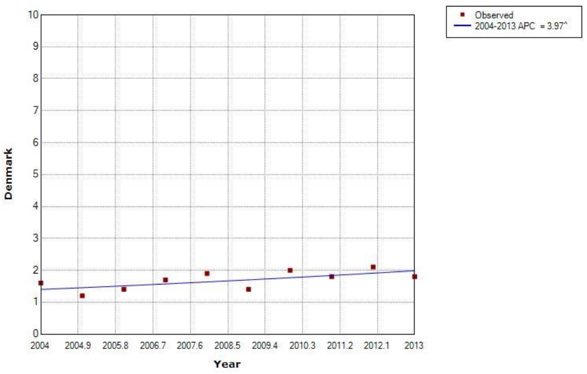
Observed
2004-2013 APC = 3.97^
Denmark
Year

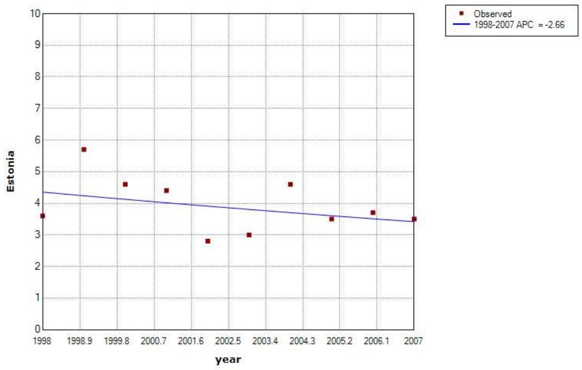
Observed
1998-2007 APC = -2.66
Estonia
year

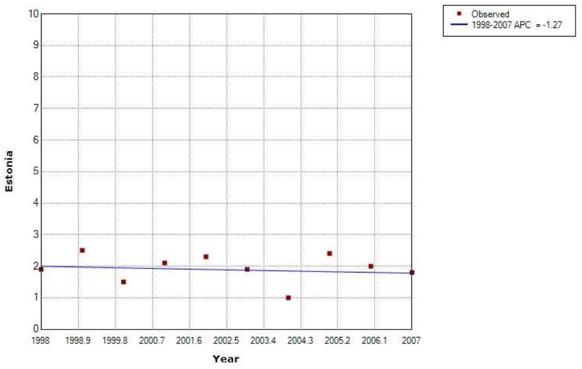
Observed
1998-2007 APC = -1.27
Estonia
Year

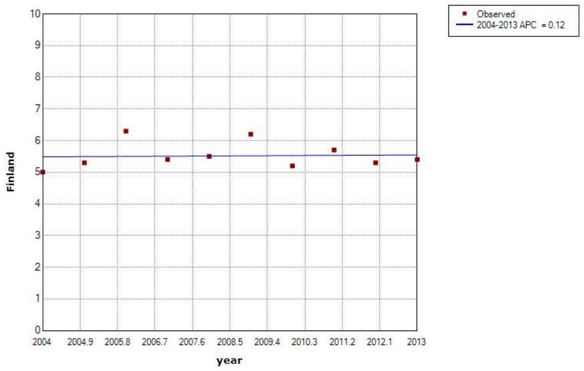
Observed
2004-2013 APC = 0.12
Finland
year

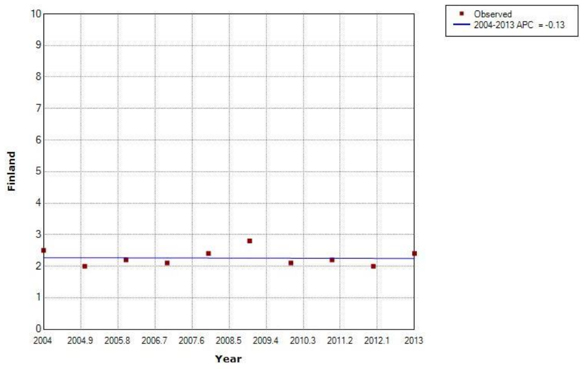
Observed
2004-2013 APC = -0.13
Finland
Year

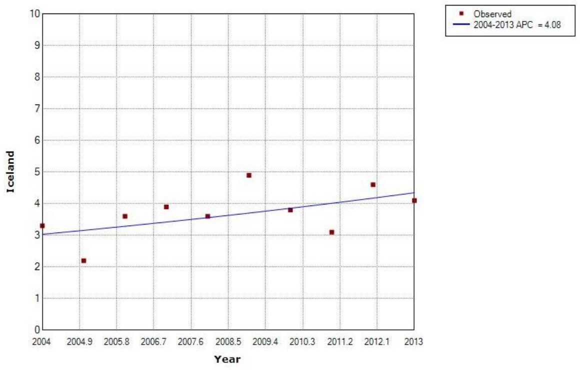
Observed
2004-2013 APC = 4.08
Iceland
Year

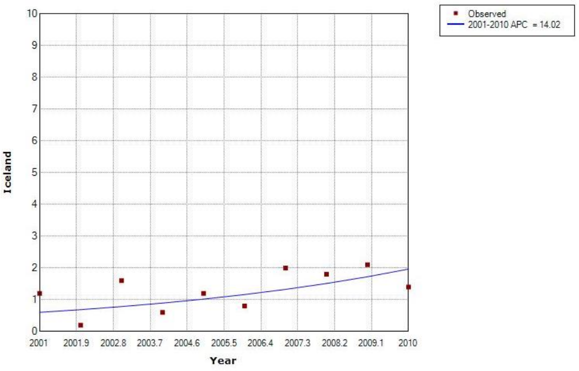
Observed
2001-2010 APC = 14.02
Iceland
Year

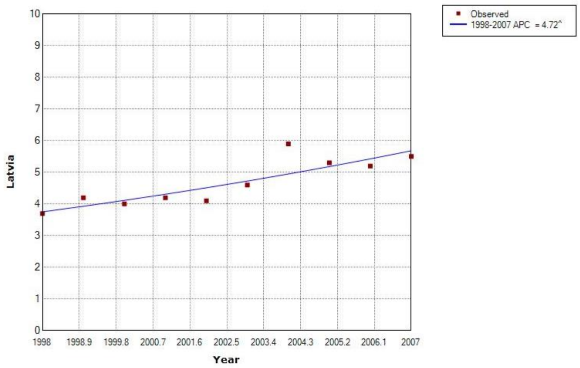
Observed
1998-2007 APC = 4.72^
Latvia
Year
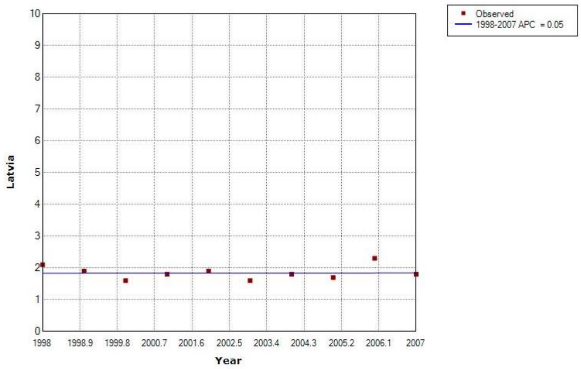
Observed
1998-2007 APC = 0.05
Latvia
Year
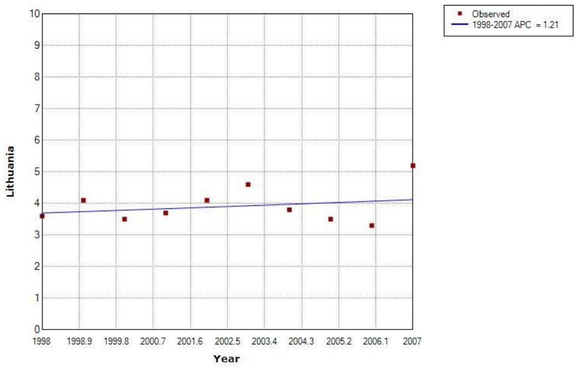
Observed
1998-2007 APC = 1.21
Lithuania
Year
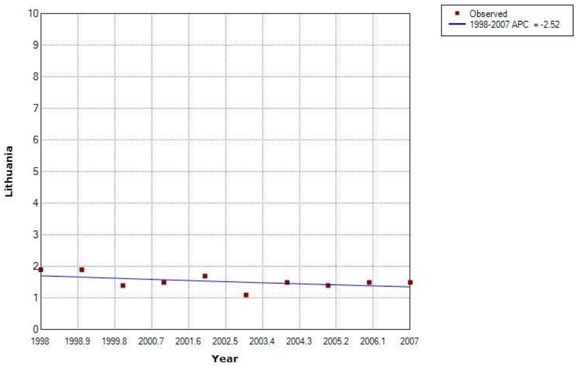
Observed
1998-2007 APC = -2.52
Lithuania
Year
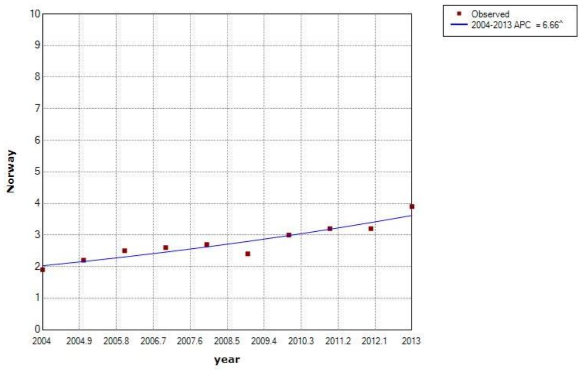
Observed
2004-2013 APC = 6.66^
Norway
year
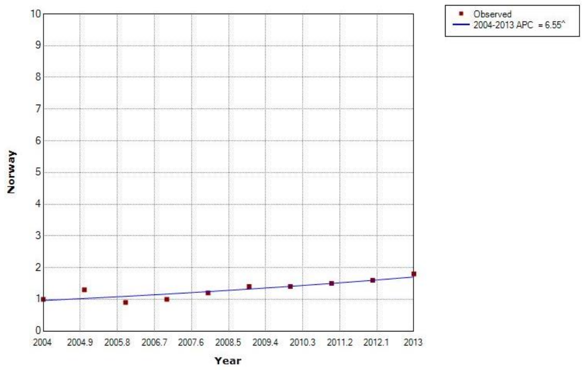
Observed
2004-2013 APC = 6.55^
Norway
Year
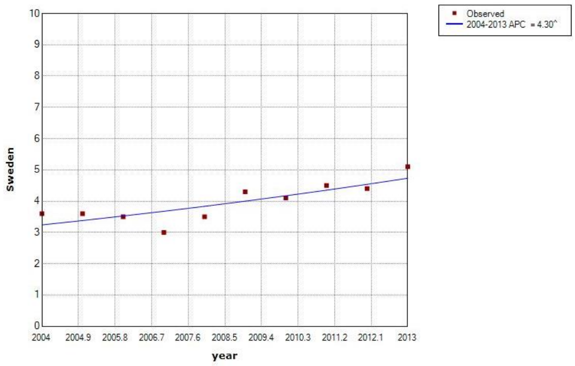
Observed
2004-2013 APC = 4.30^
Sweden
year
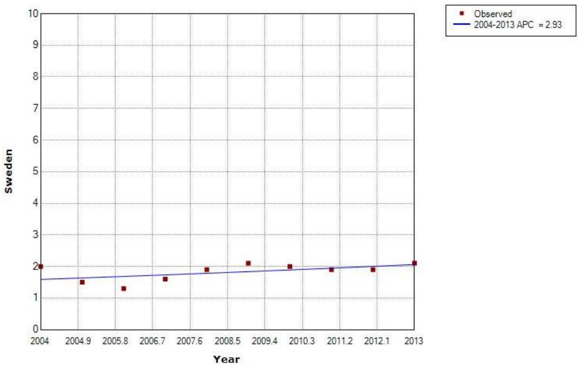
Observed
2004-2013 APC = 2.93
Sweden
Year

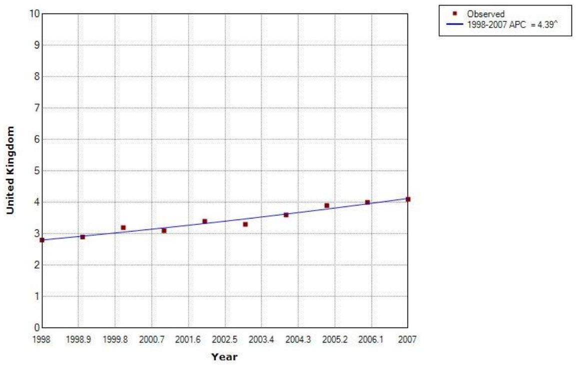

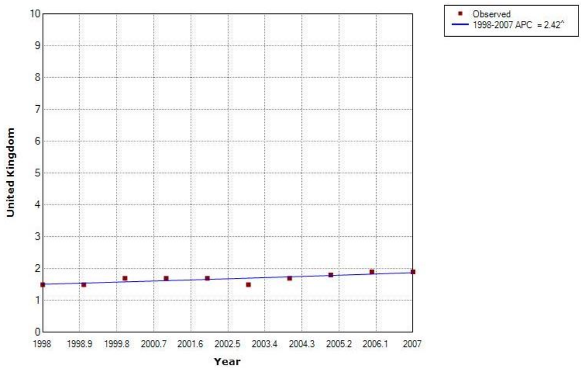

5) Western Europe

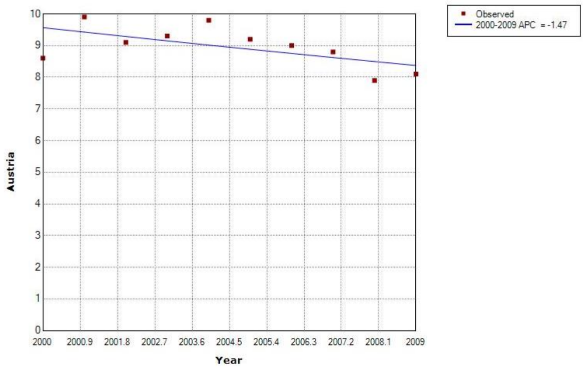

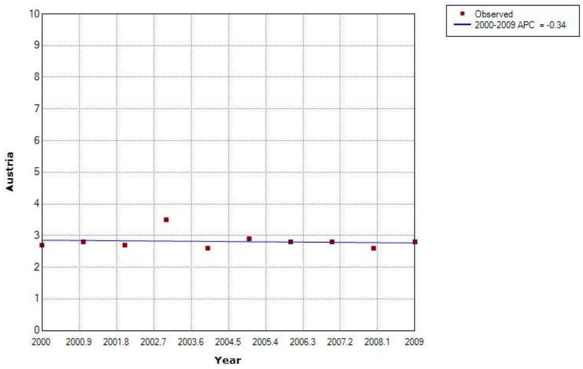

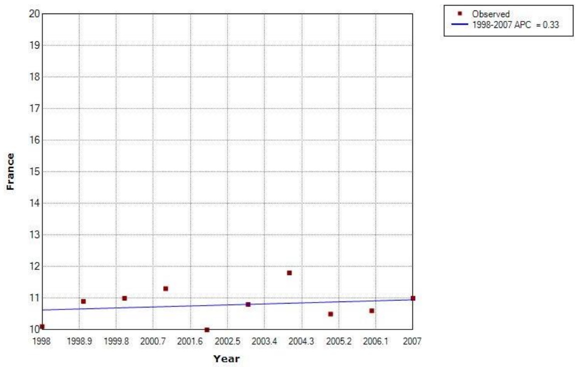

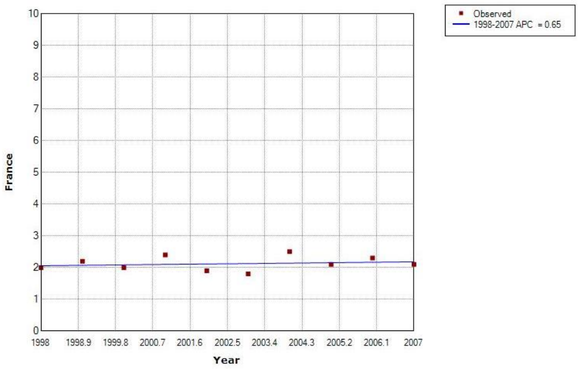

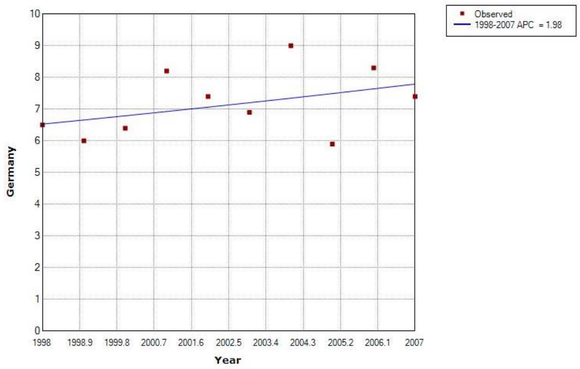

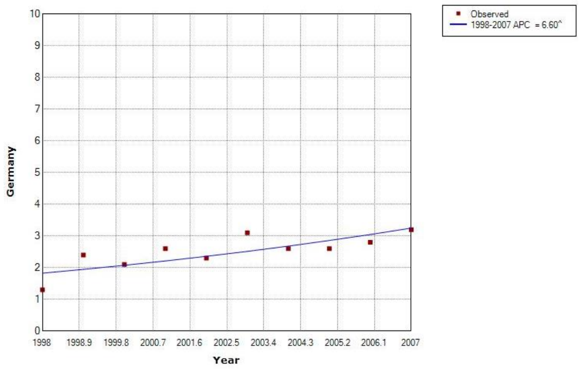

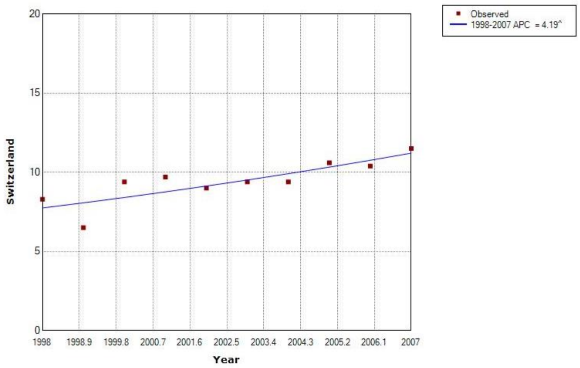

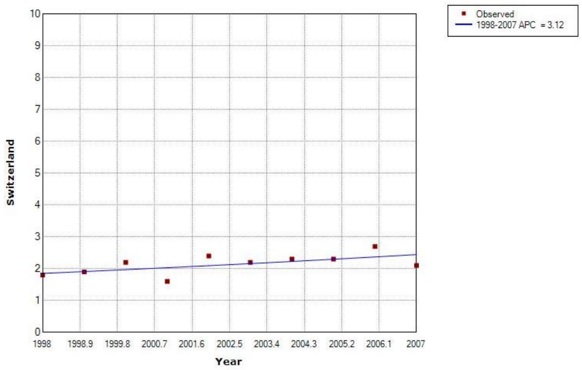

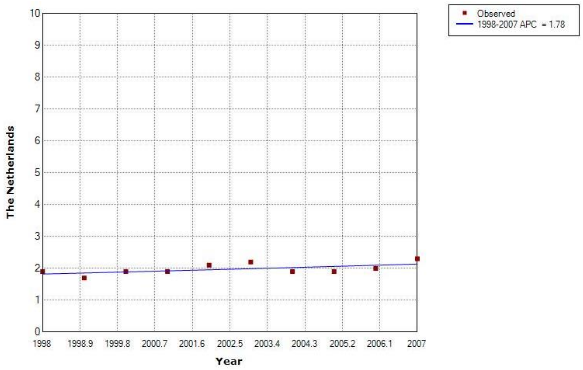

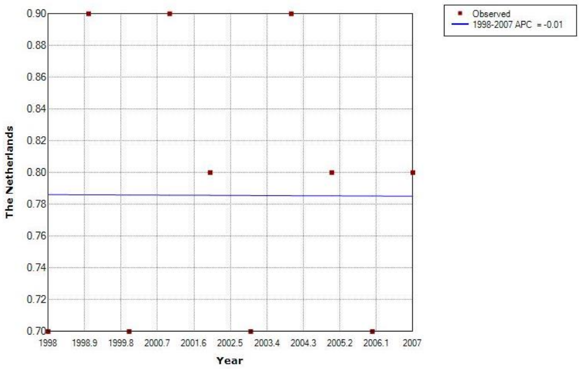

6) Southern Europe

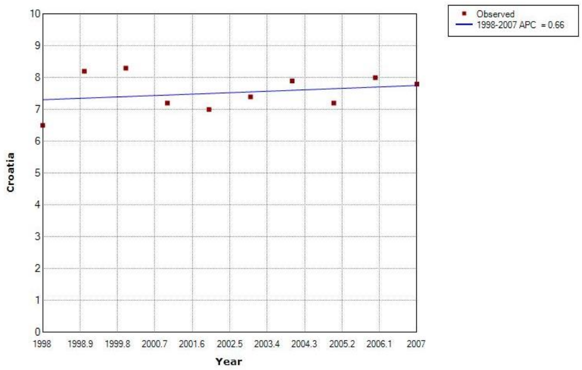

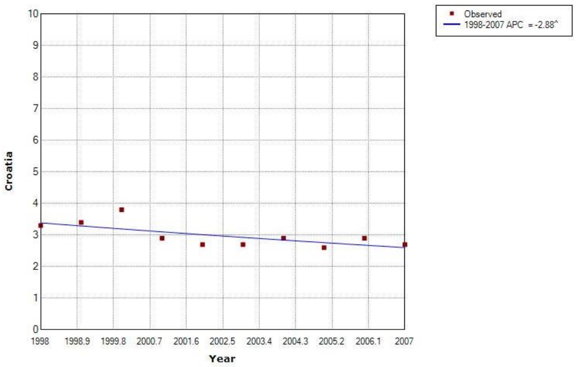

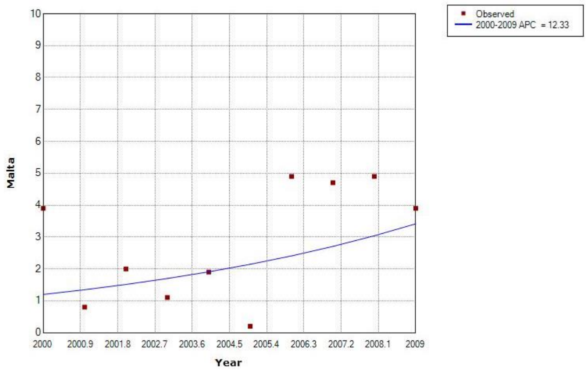

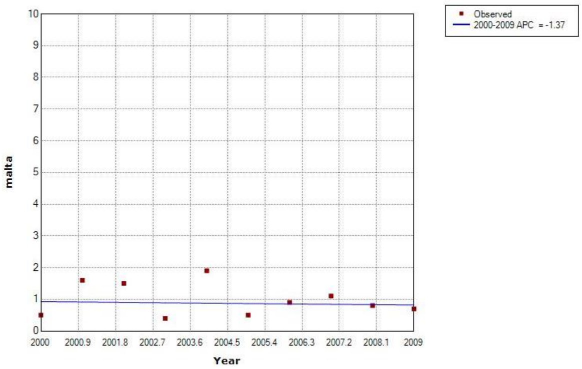

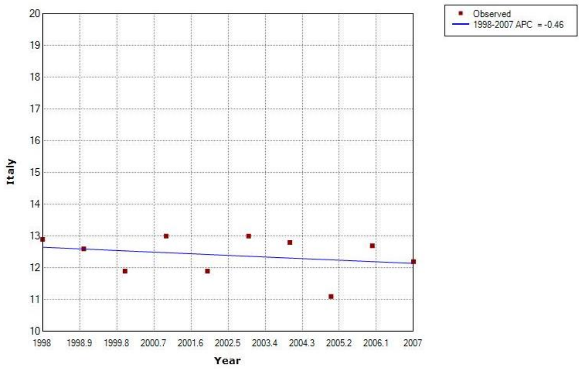

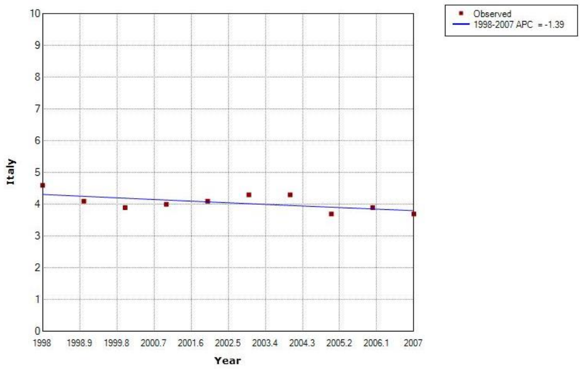

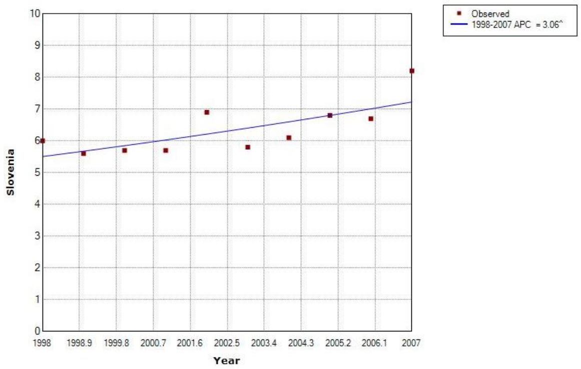

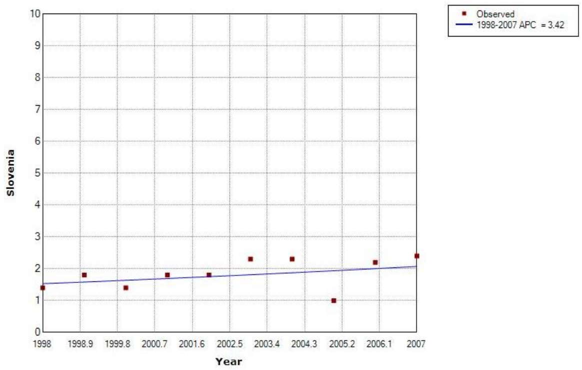

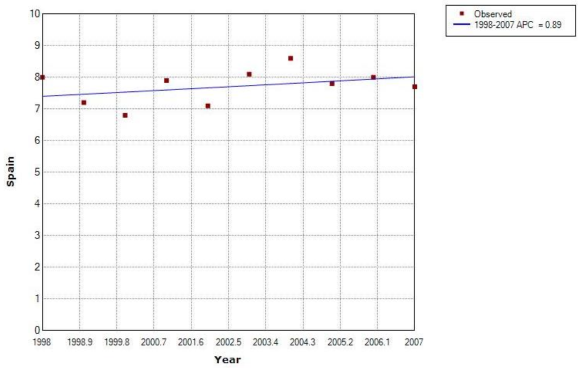

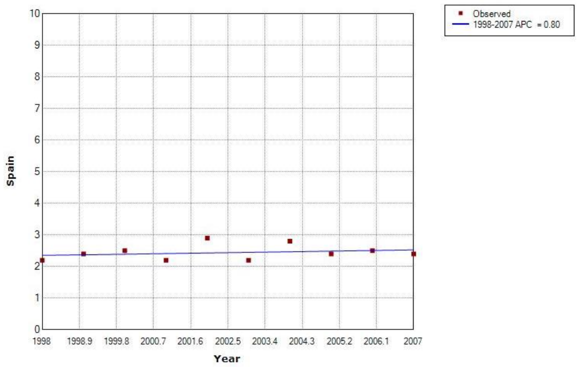

7) Eastern Europe

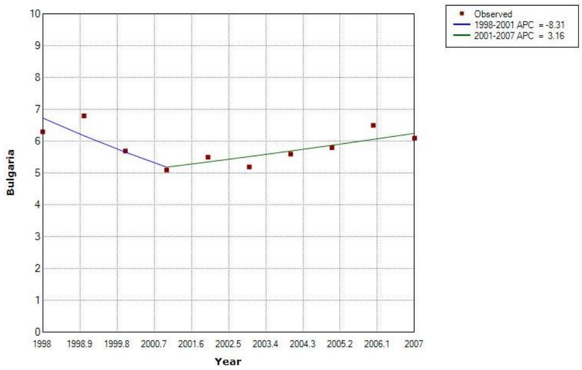

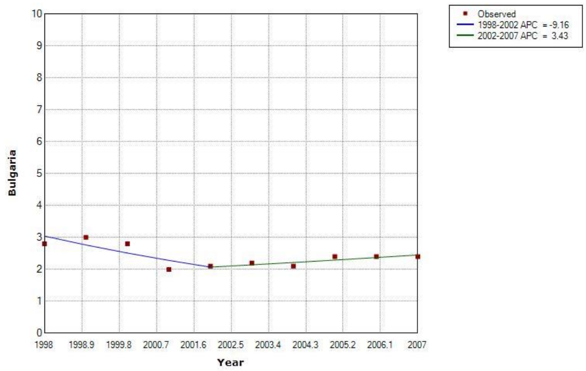

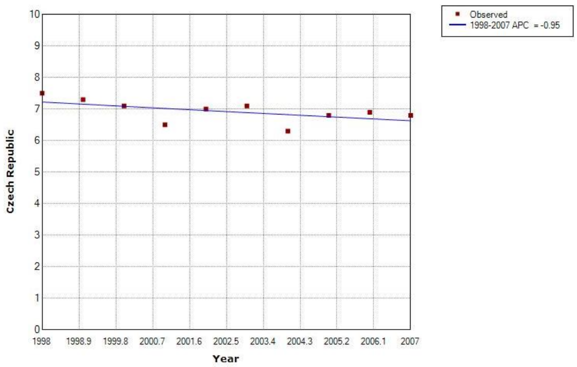
Observed
1998-2007 APC = -0.95
Czech Republic
Year

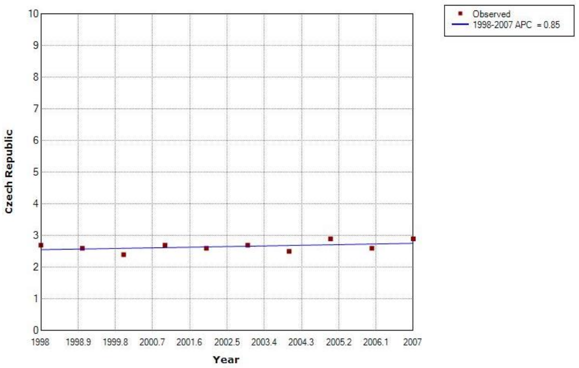
Observed
1998-2007 APC = 0.85
Czech Republic
Year

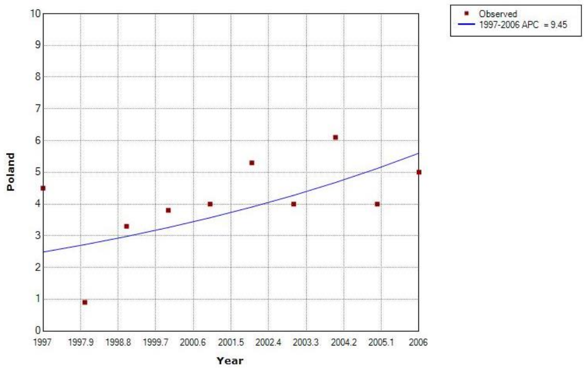
Observed
1997-2006 APC = 9.45
Poland
Year

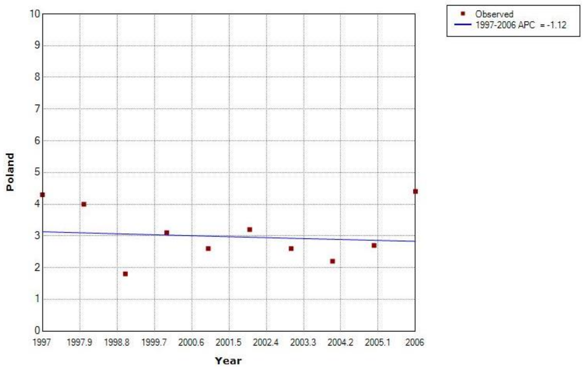
Observed
1997-2006 APC = -1.12
Poland
Year

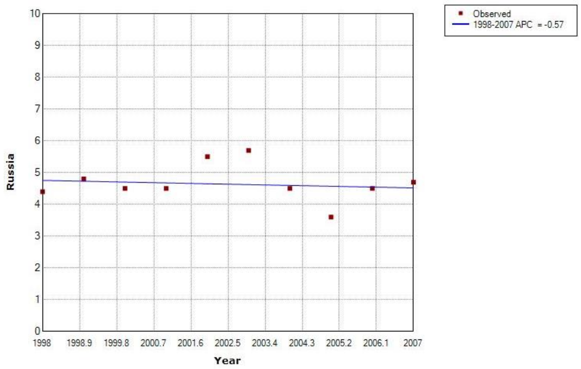
Observed
1998-2007 APC = -0.57
Russia
Year

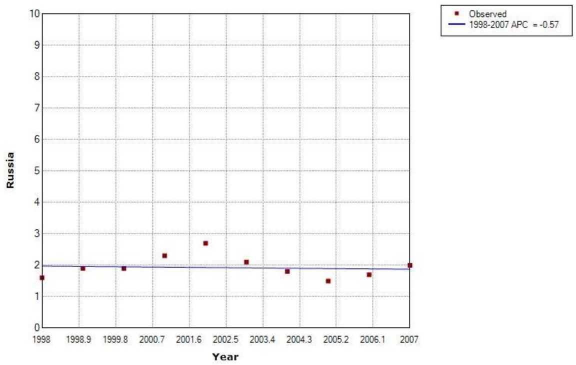
Observed
1998-2007 APC = -0.57
Russia
Year

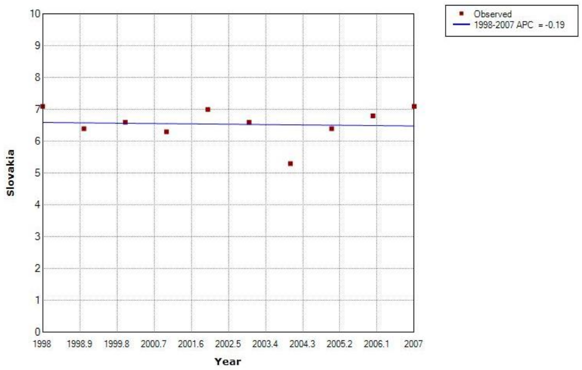
Observed
1998-2007 APC = -0.19
Slovakia
Year

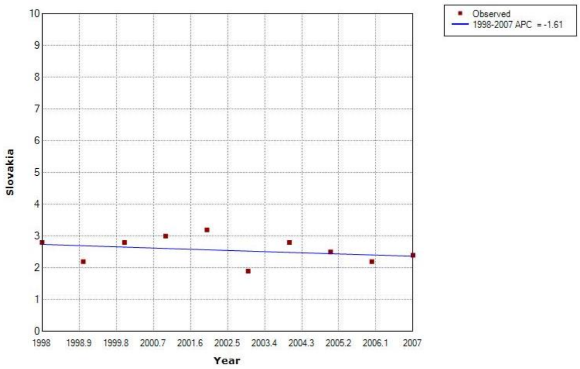
Observed
1998-2007 APC = -1.61
Slovakia
Year

Supplementary Figure 3 Findings from the joinpoint regression analysis of the global mortality rates of liver cancer (Left: Male, Right: Female)

1) Latin America and the Caribbean

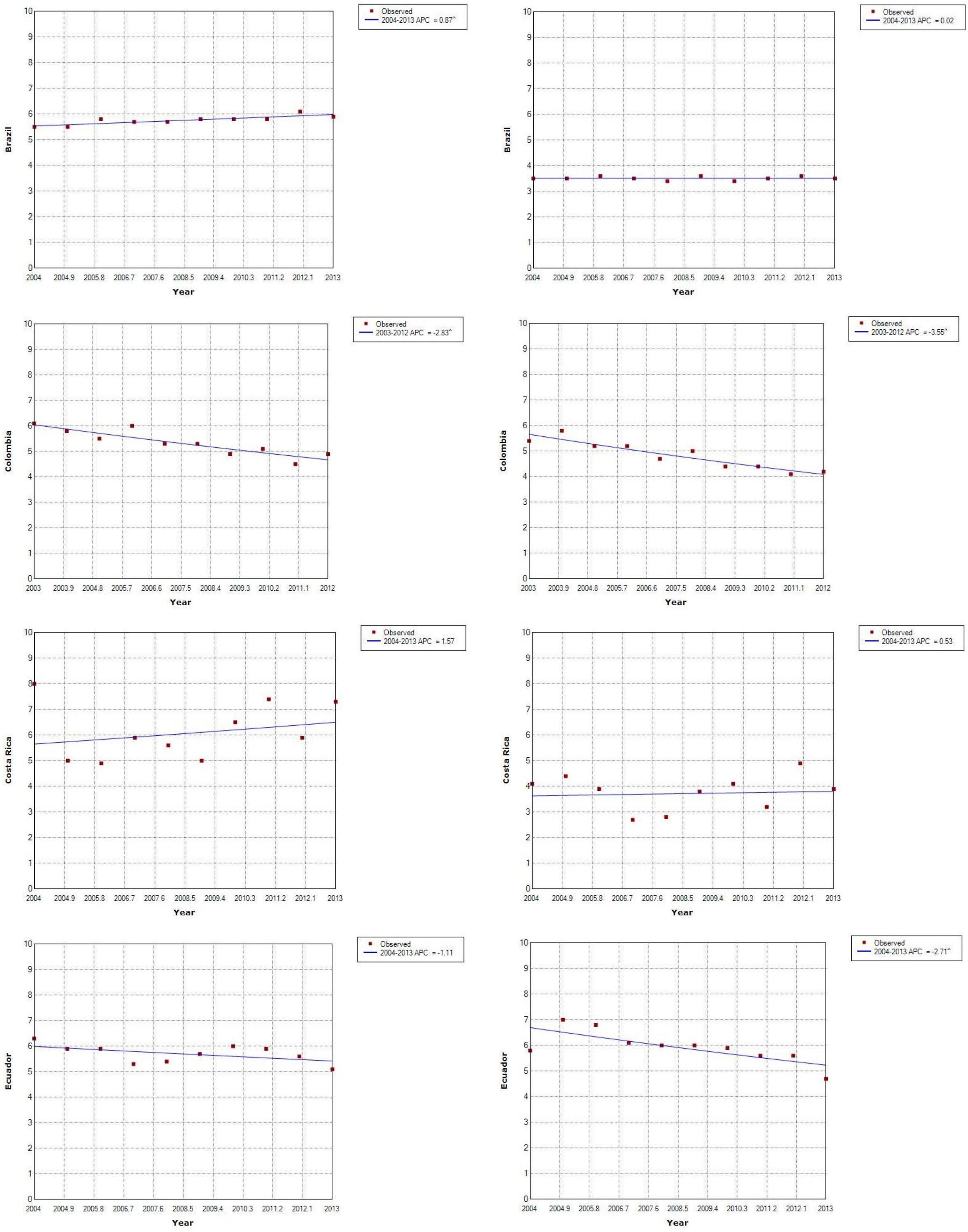

2) Northern America

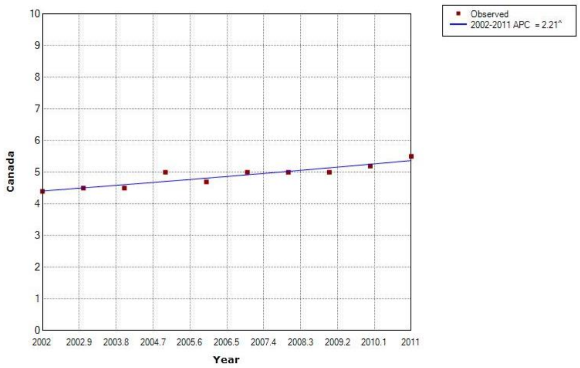
Canada
Year
2002 2002.9 2003.8 2004.7 2005.6 2006.5 2007.4 2008.3 2009.2 2010.1 2011
Observed
2002-2011 APC = 2.21^

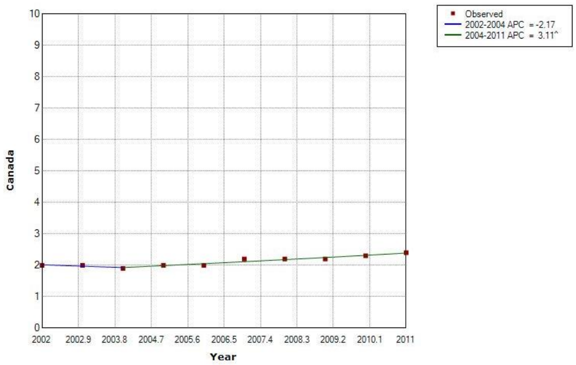
Canada
Year
2002 2002.9 2003.8 2004.7 2005.6 2006.5 2007.4 2008.3 2009.2 2010.1 2011
Observed
2002-2004 APC = -2.17
2004-2011 APC = 3.11^

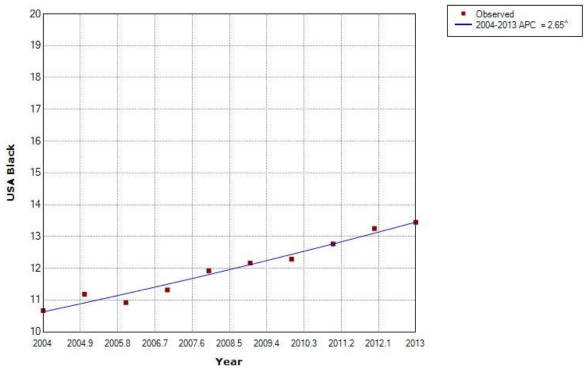
USA Black
Year
2004 2004.9 2005.8 2006.7 2007.6 2008.5 2009.4 2010.3 2011.2 2012.1 2013
Observed
2004-2013 APC = 2.65^

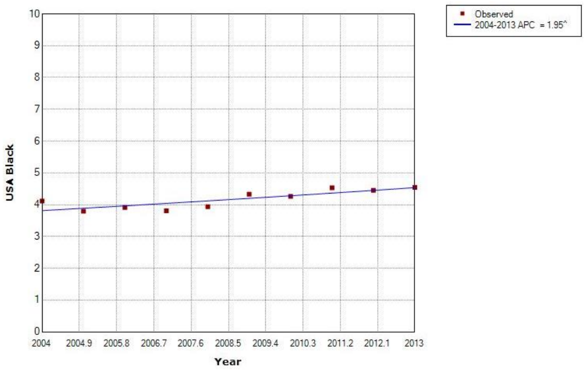
USA Black
Year
2004 2004.9 2005.8 2006.7 2007.6 2008.5 2009.4 2010.3 2011.2 2012.1 2013
Observed
2004-2013 APC = 1.95^

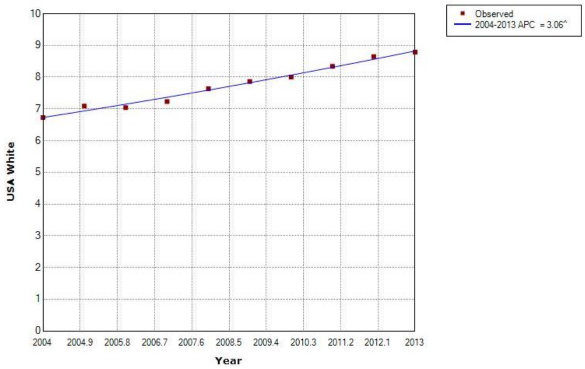
USA White
Year
2004 2004.9 2005.8 2006.7 2007.6 2008.5 2009.4 2010.3 2011.2 2012.1 2013
Observed
2004-2013 APC = 3.06^

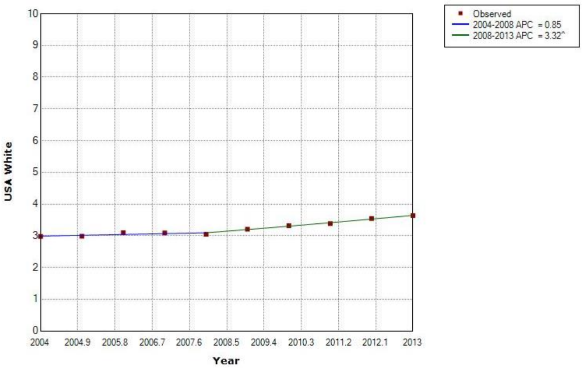
USA White
Year
2004 2004.9 2005.8 2006.7 2007.6 2008.5 2009.4 2010.3 2011.2 2012.1 2013
Observed
2004-2008 APC = 0.85
2008-2013 APC = 3.32^

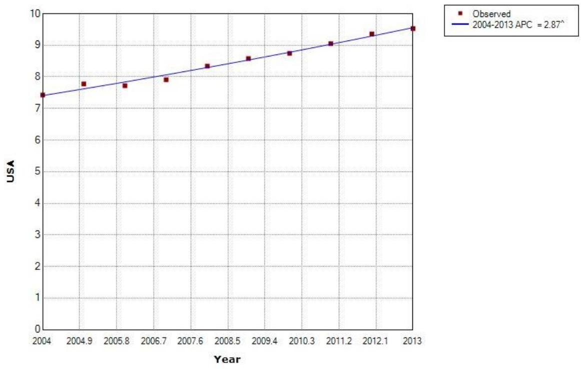
USA
Year
2004 2004.9 2005.8 2006.7 2007.6 2008.5 2009.4 2010.3 2011.2 2012.1 2013
Observed
2004-2013 APC = 2.87^

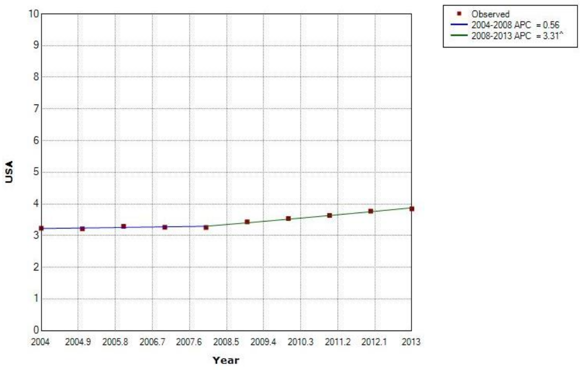
USA
Year
2004 2004.9 2005.8 2006.7 2007.6 2008.5 2009.4 2010.3 2011.2 2012.1 2013
Observed
2004-2008 APC = 0.56
2008-2013 APC = 3.31^

3) Asia

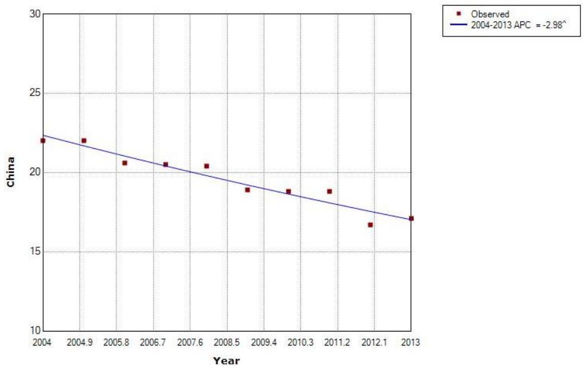
Observed
2004-2013 APC = -2.98^
China
Year

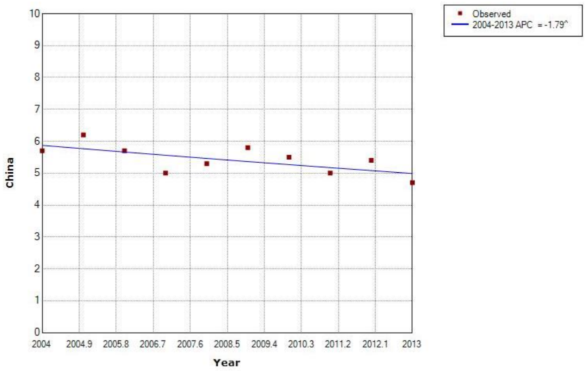
Observed
2004-2013 APC = -1.79^
China
Year

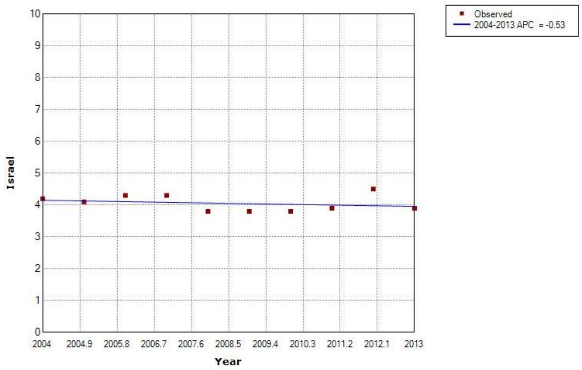
Observed
2004-2013 APC = -0.53
Israel
Year

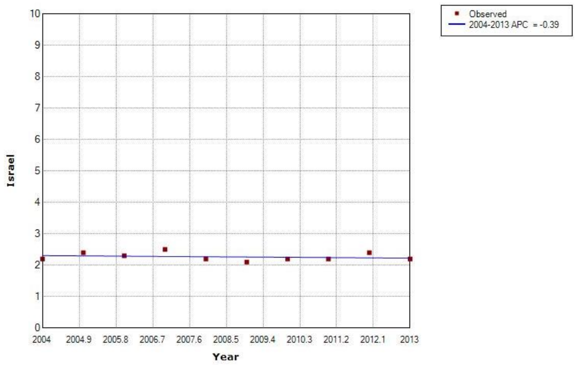
Observed
2004-2013 APC = -0.39
Israel
Year

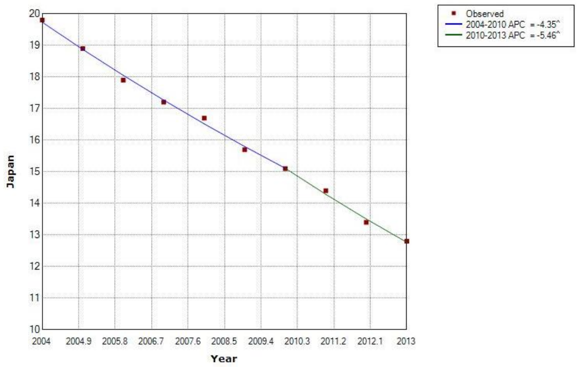
Observed
2004-2010 APC = -4.35^
2010-2013 APC = -5.46^
Japan
Year

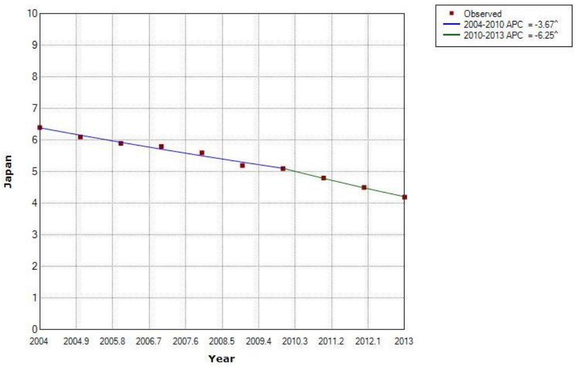
Observed
2004-2010 APC = -3.67^
2010-2013 APC = -6.25^
Japan
Year

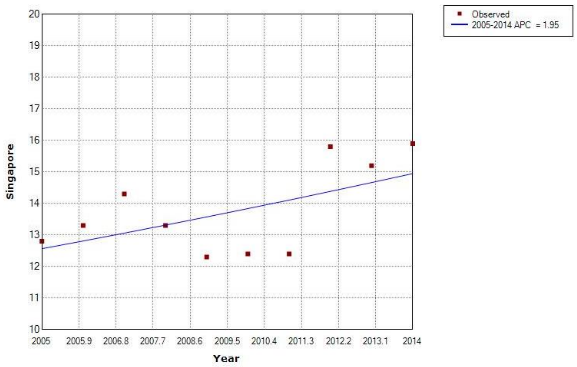
Observed
2005-2014 APC = 1.95
Singapore
Year

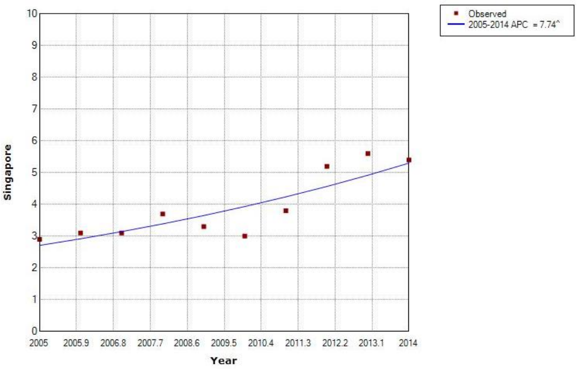
Observed
2005-2014 APC = 7.74^
Singapore
Year

4) Oceania

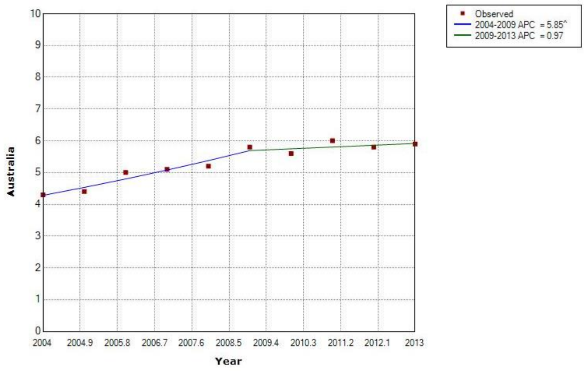

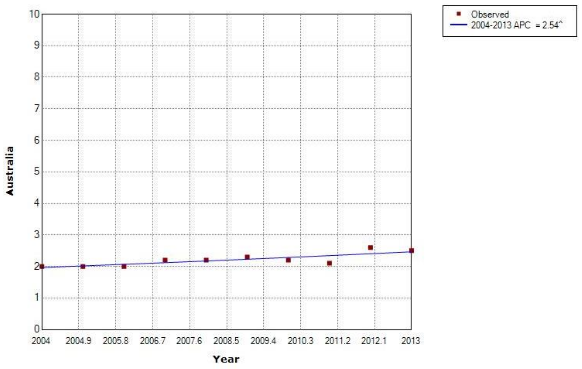

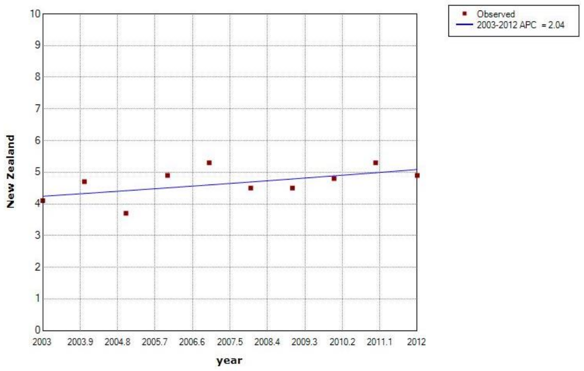

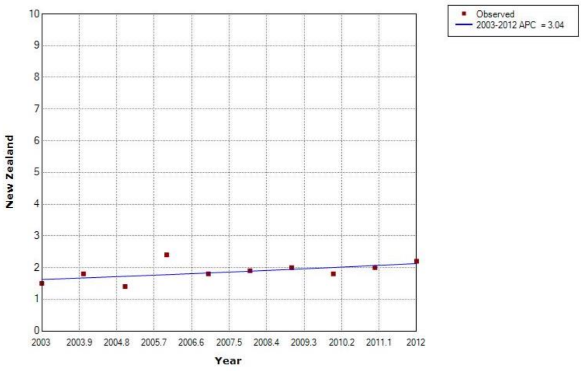

5) Northern Europe

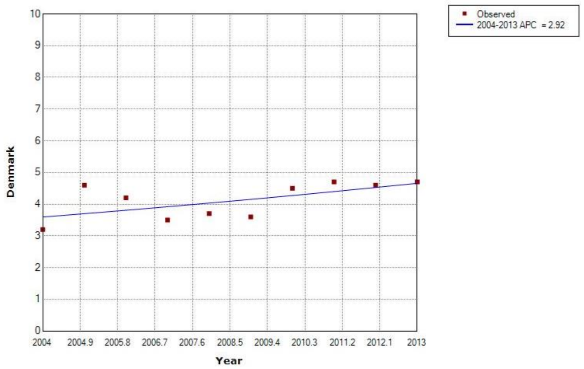

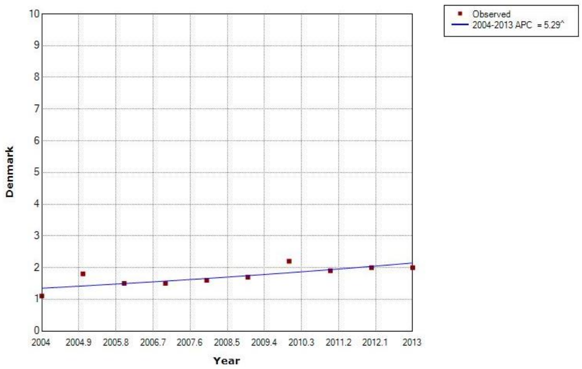

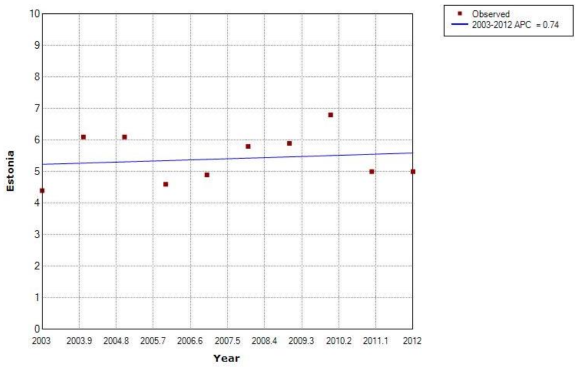

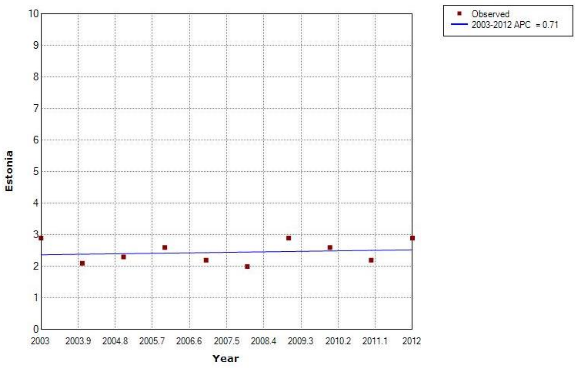

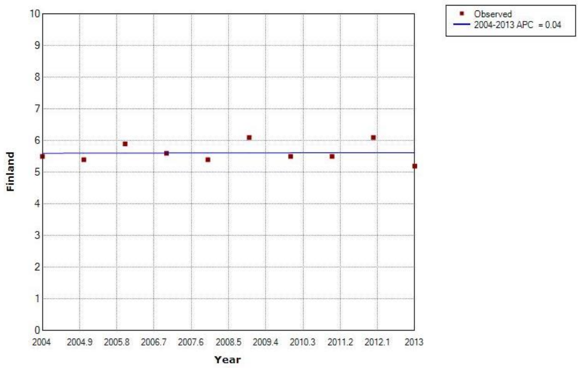
Observed
2004-2013 APC = 0.04
Finland
Year

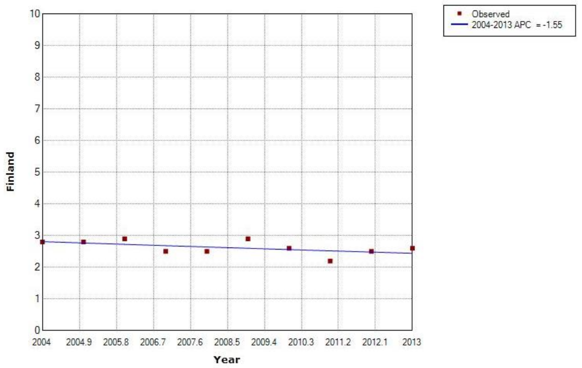
Observed
2004-2013 APC = -1.55
Finland
Year

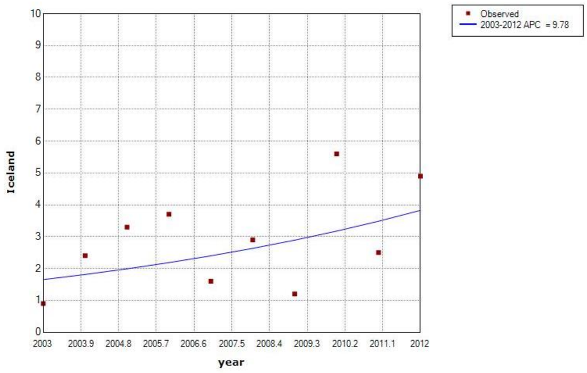
Observed
2003-2012 APC = 9.78
Iceland
year

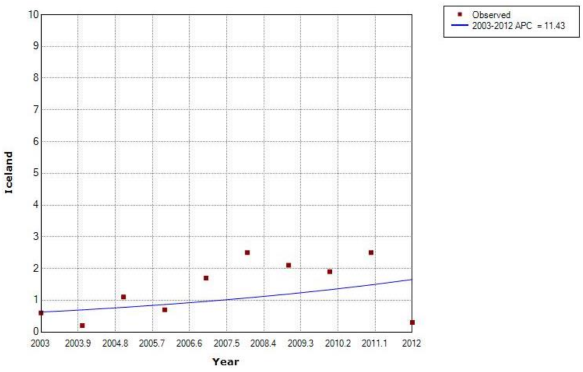
Observed
2003-2012 APC = 11.43
Iceland
Year

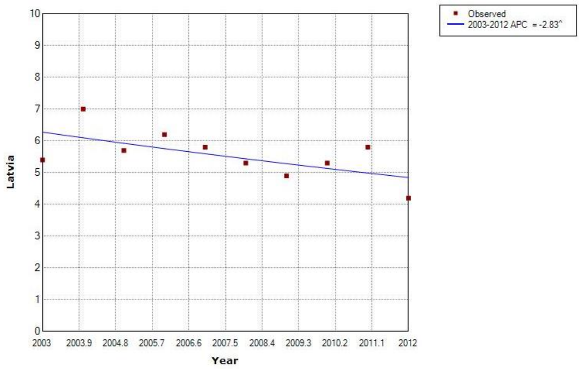
Observed
2003-2012 APC = -2.83^
Latvia
Year

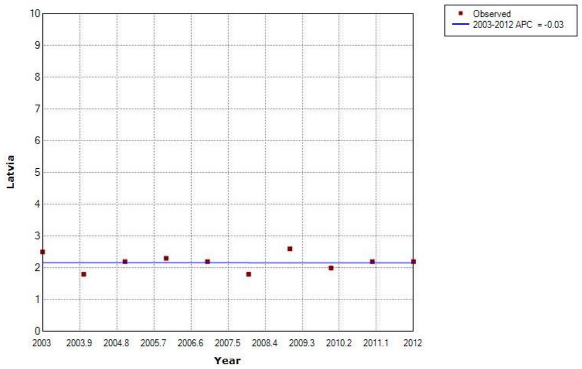
Observed
2003-2012 APC = -0.03
Latvia
Year

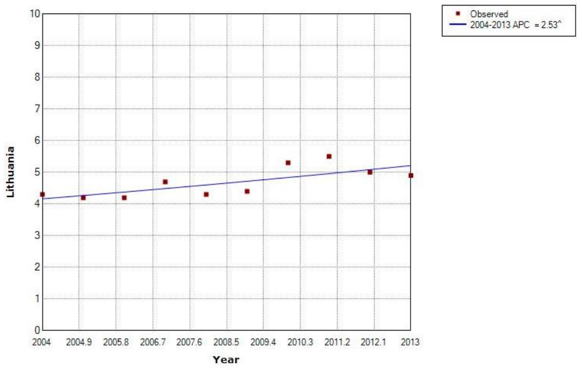
Observed
2004-2013 APC = 2.53^
Lithuania
Year

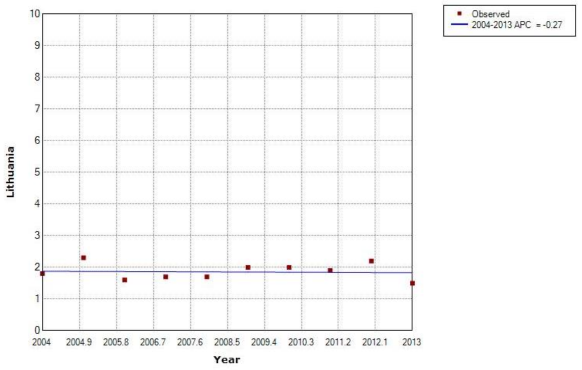
Observed
2004-2013 APC = -0.27
Lithuania
Year

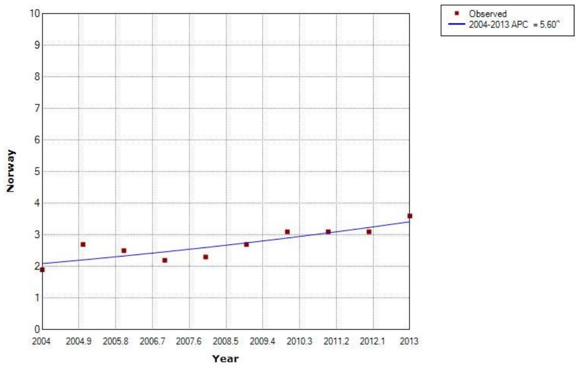

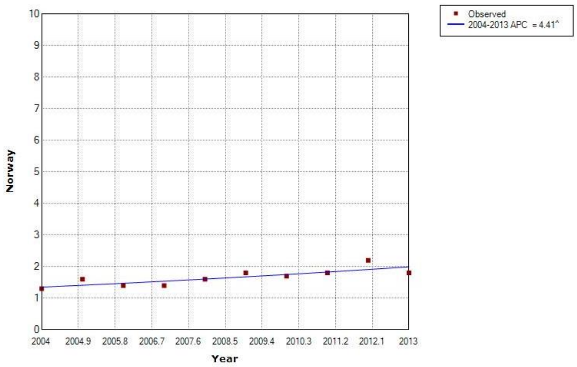

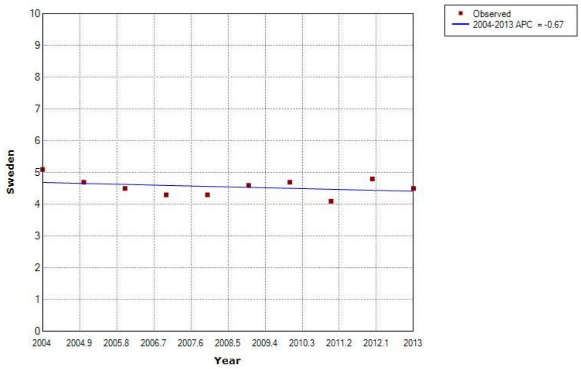

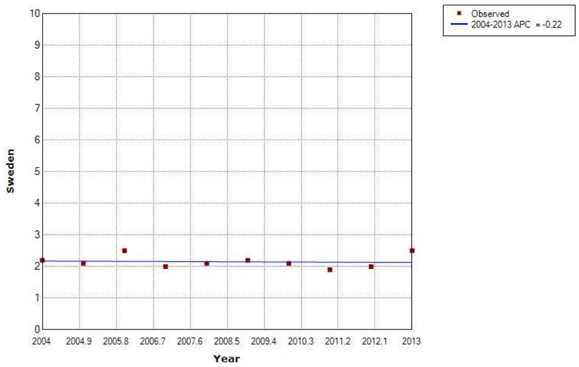

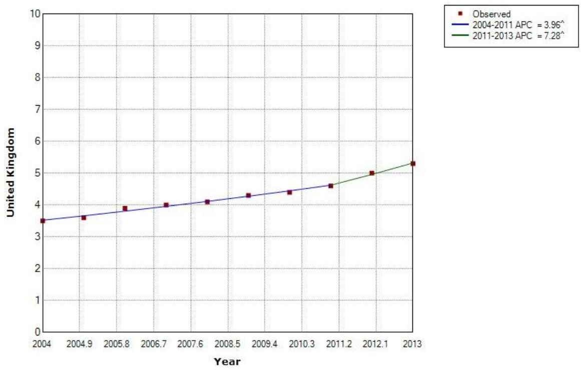

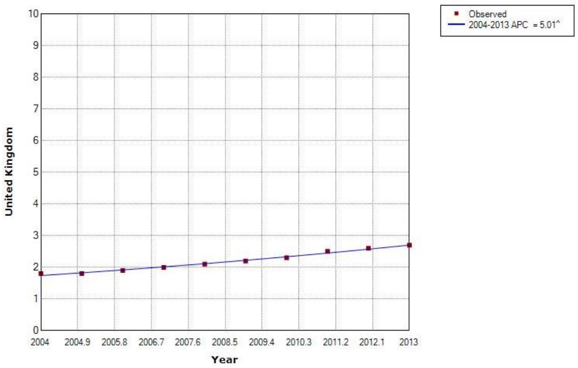

6) Western Europe

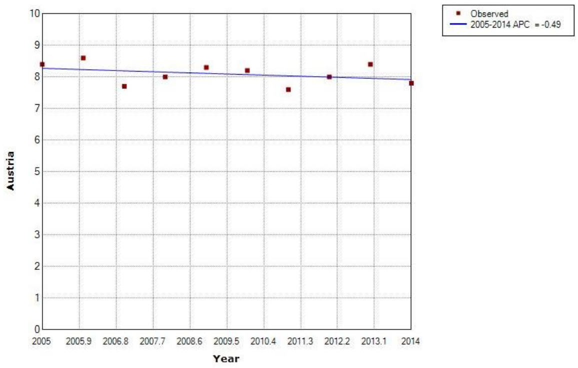

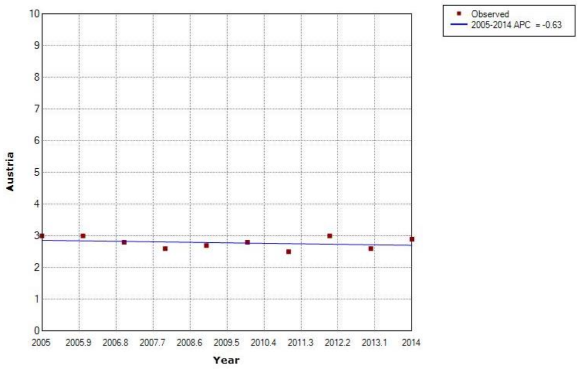

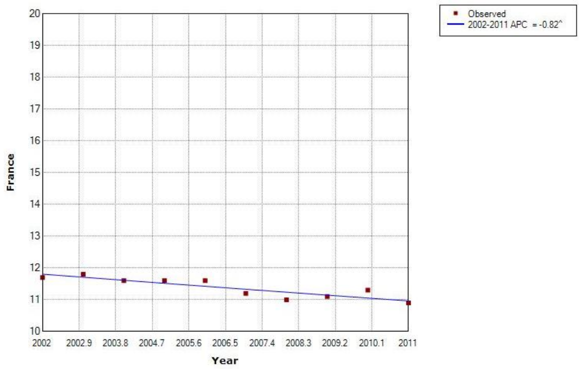
Observed
2002-2011 APC = -0.82^
France
Year

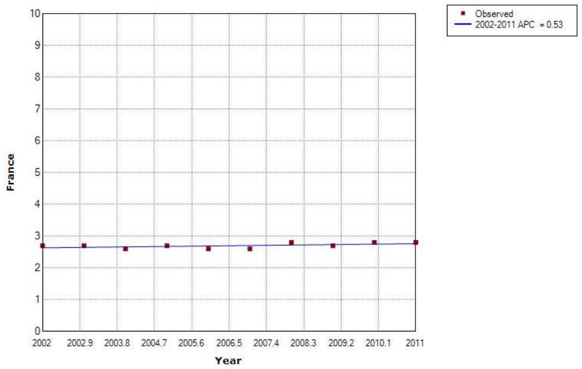
Observed
2002-2011 APC = 0.53
France
Year

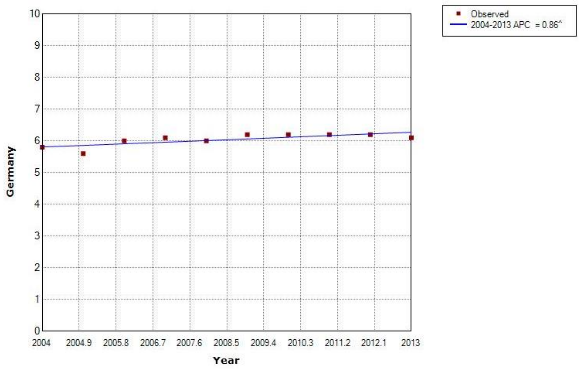
Observed
2004-2013 APC = 0.86^
Germany
Year

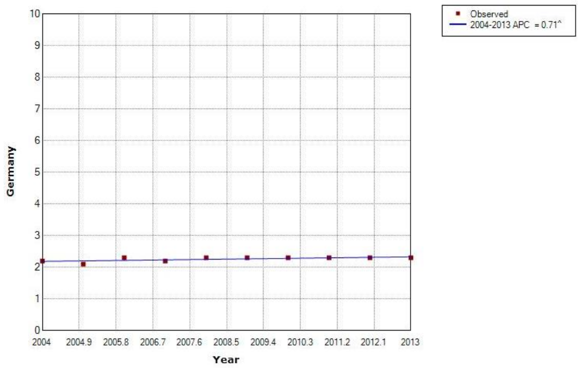
Observed
2004-2013 APC = 0.71^
Germany
Year

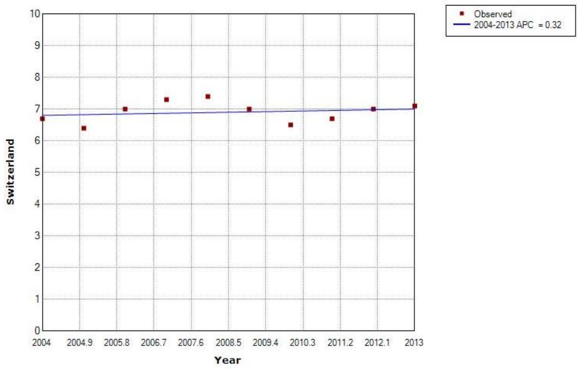
Observed
2004-2013 APC = 0.32
Switzerland
Year

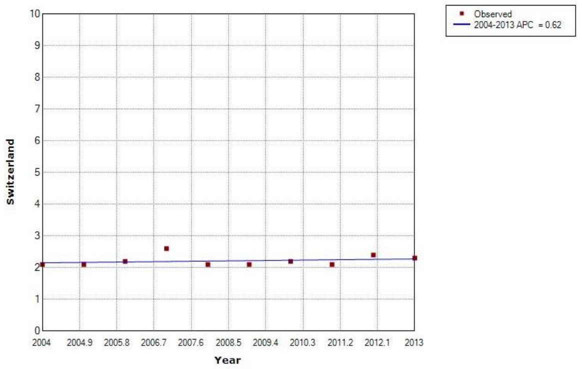
Observed
2004-2013 APC = 0.62
Switzerland
Year

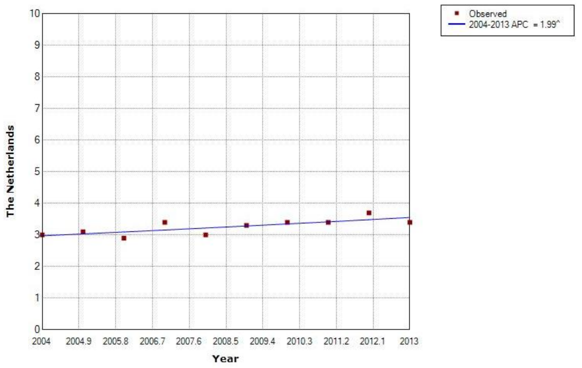
Observed
2004-2013 APC = 1.99^
The Netherlands
Year

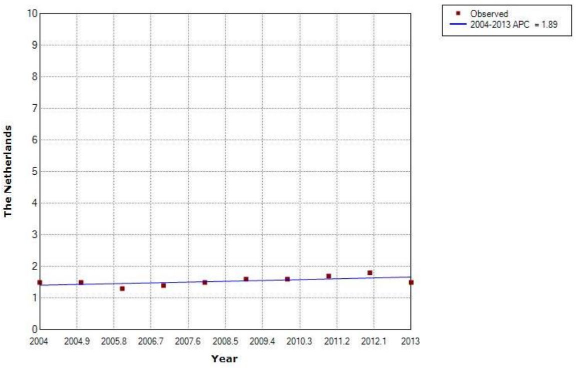
Observed
2004-2013 APC = 1.89
The Netherlands
Year

7) Southern Europe

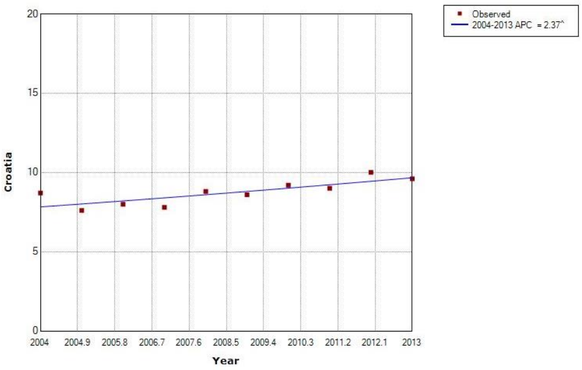

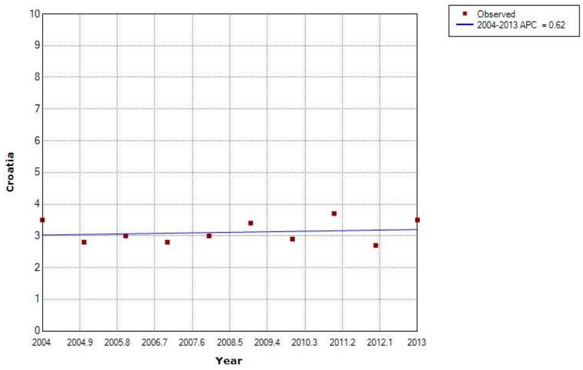

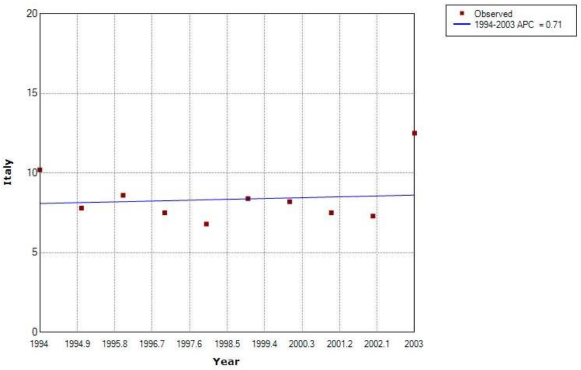

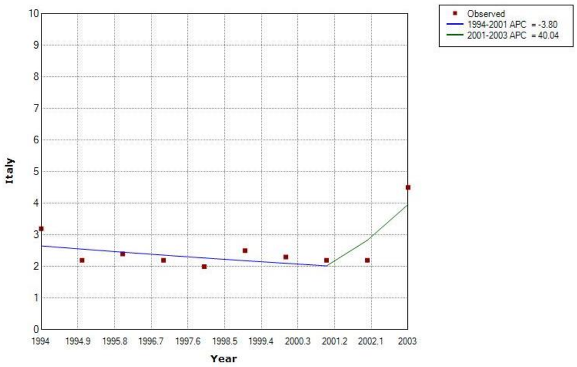

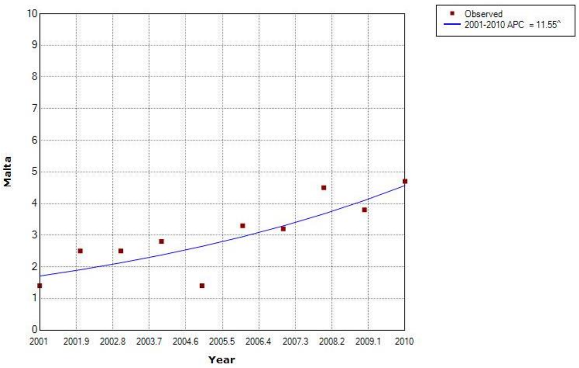

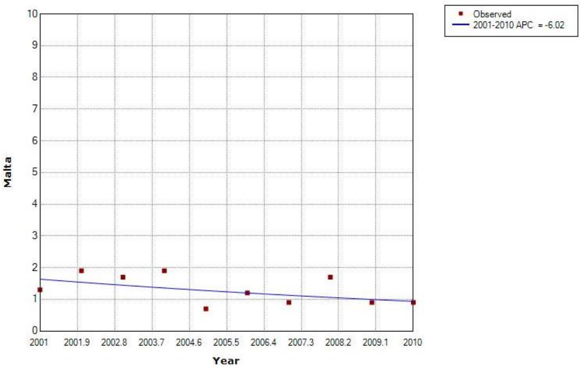

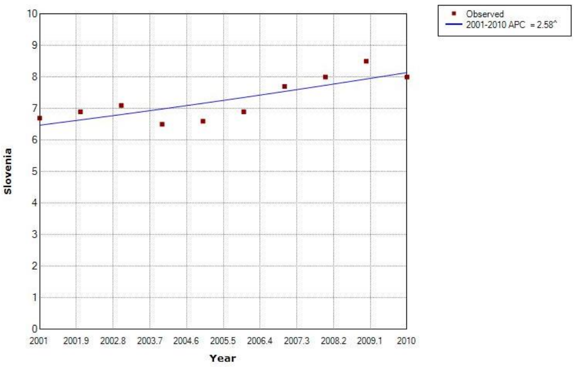

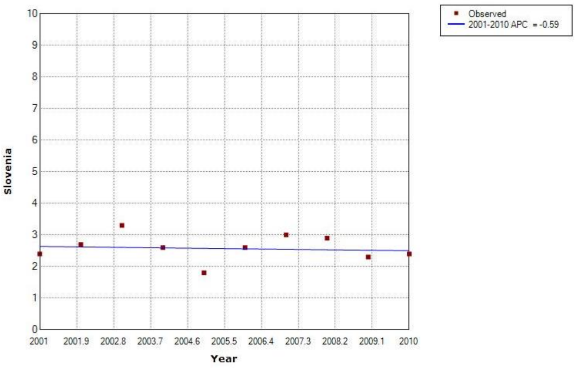

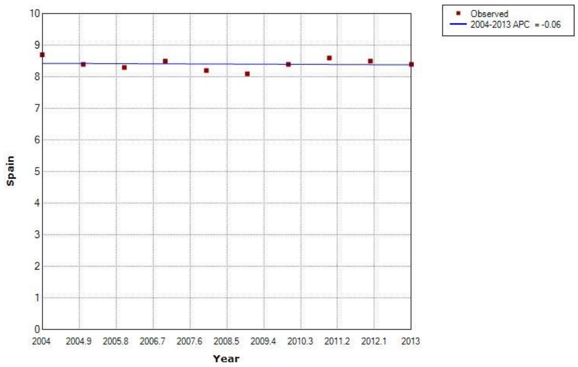

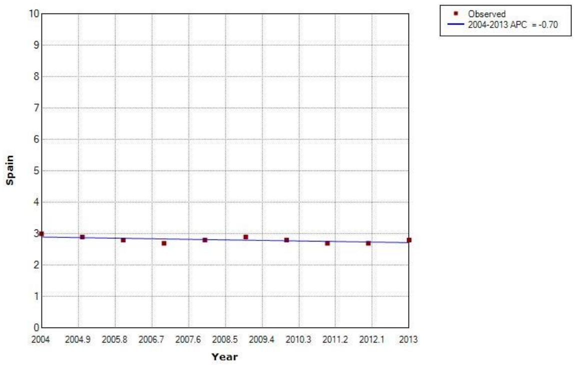

8) Eastern Europe

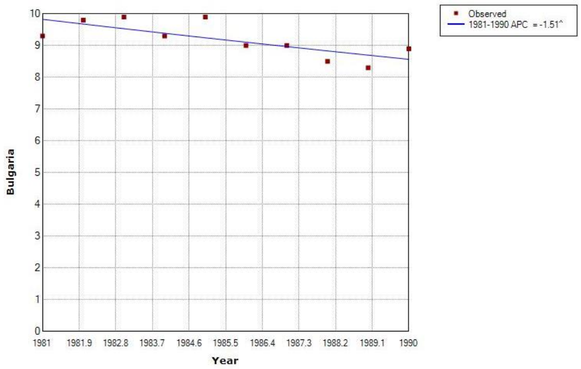

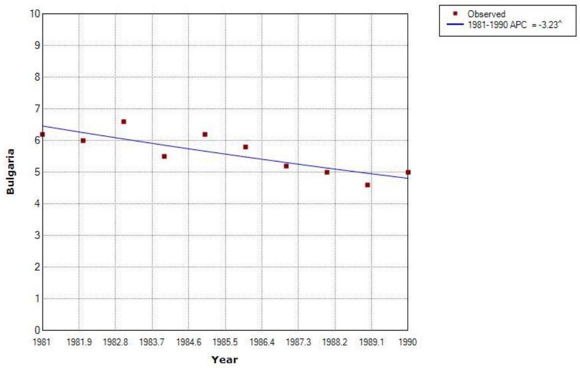

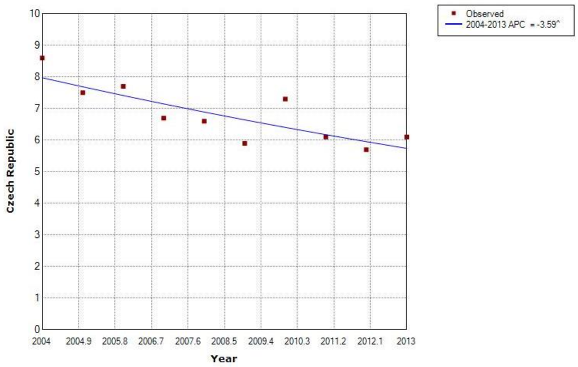

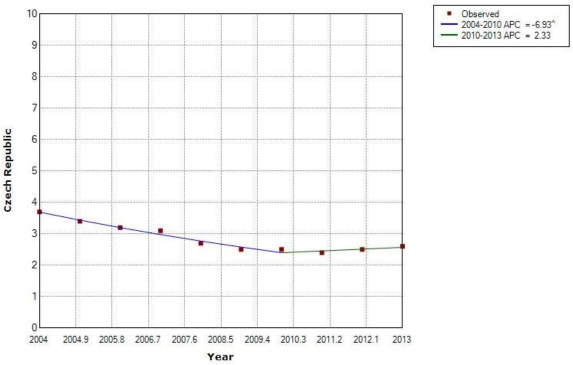

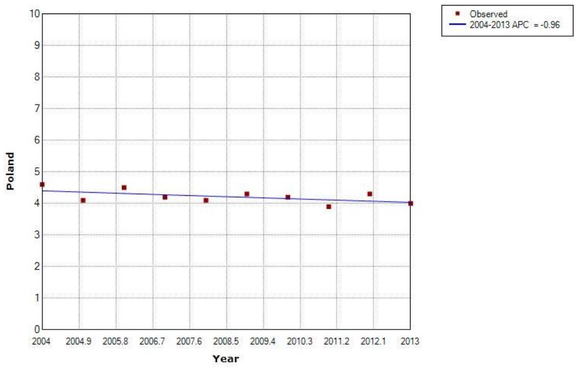

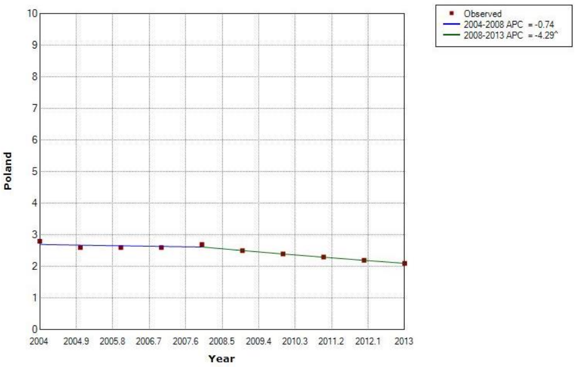

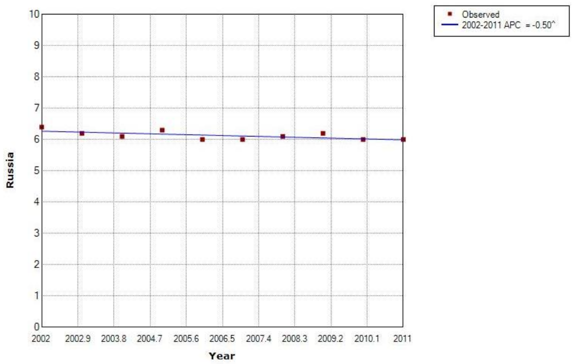
Observed
2002-2011 APC = -0.50^
Russia
Year
0
1
2
3
4
5
6
7
8
9
10
2002
2002.9
2003.8
2004.7
2005.6
2006.5
2007.4
2008.3
2009.2
2010.1
2011

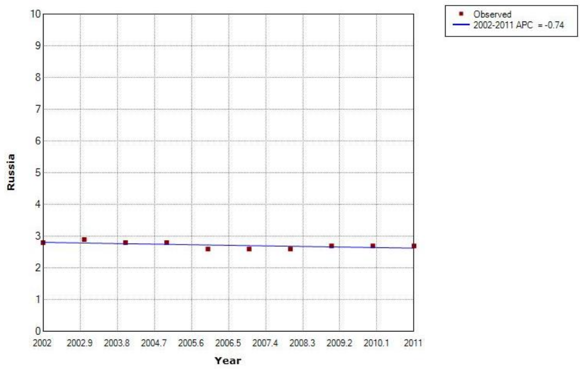
Observed
2002-2011 APC = -0.74
Russia
Year
0
1
2
3
4
5
6
7
8
9
10
2002
2002.9
2003.8
2004.7
2005.6
2006.5
2007.4
2008.3
2009.2
2010.1
2011

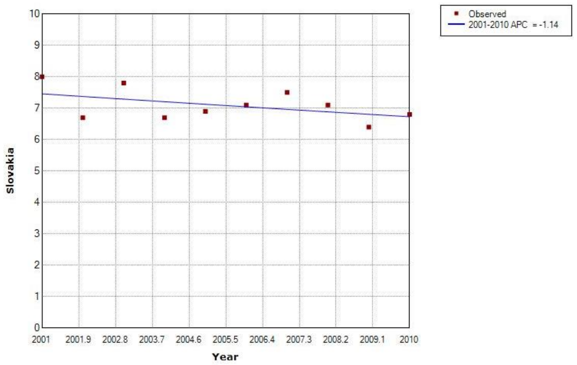
Observed
2001-2010 APC = -1.14
Slovakia
Year
0
1
2
3
4
5
6
7
8
9
10
2001
2001.9
2002.8
2003.7
2004.6
2005.5
2006.4
2007.3
2008.2
2009.1
2010

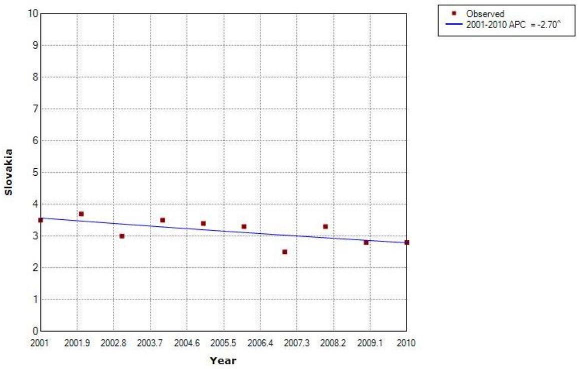
Observed
2001-2010 APC = -2.70^
Slovakia
Year
0
1
2
3
4
5
6
7
8
9
10
2001
2001.9
2002.8
2003.7
2004.6
2005.5
2006.4
2007.3
2008.2
2009.1
2010
